# How competition between overlapping generations can influence optimal egg-laying strategies in annual social insects

**DOI:** 10.1007/s00442-023-05411-z

**Published:** 2023-07-10

**Authors:** Jacob Johansson, Andres N. Arce, Richard J. Gill

**Affiliations:** 1 Department of Biology, Lund University, Sölvegatan 37, 22362 Lund, Sweden; 2 Department of Life Sciences, Georgina Mace Centre for the Living Planet, Imperial College London, Silwood Park Campus, Buckhurst Road, Ascot, Berkshire SL5 7PY UK; 3 School of Engineering, Arts, Science and Technology, University of Suffolk, 19 Neptune Quay, Ipswich, IP4 1QJ UK

**Keywords:** Colony dynamics, Number-size trade-offs, Sexuals, Social insects, Workers

## Abstract

Annual social insects are an integral functional group of organisms, particularly in temperate environments. An emblematic part of their annual cycle is the social phase, during which the colony-founding queen rears workers that later assist her in rearing sexual progeny (gynes and drones). In many annual social insects, such as species of bees, wasps, and other groups, developing larvae are provisioned gradually as they develop (progressive provisioning) leading to multiple larval generations being reared simultaneously. We present a model for how the queen in such cases should optimize her egg-laying rate throughout the social phase depending on number-size trade-offs, colony age-structure, and energy balance. Complementing previous theory on optimal allocation between workers vs. sexuals in annual social insects and on temporal egg-laying patterns in solitary insects, we elucidate how resource competition among overlapping larval generations can influence optimal egg-laying strategies. With model parameters informed by knowledge of a common bumblebee species, the optimal egg-laying schedule consists of two temporally separated early broods followed by a more continuous rearing phase, matching empirical observations. However, eggs should initially be laid continuously at a gradually increasing rate when resources are scarce or mortality risks high and in cases where larvae are fully supplied with resources at the egg-laying stage (mass-provisioning). These factors, alongside sexual:worker body size ratios, further determine the overall trend in egg-laying rates over the colony cycle. Our analysis provides an inroad to study and mechanistically understand variation in colony development strategies within and across species of annual social insects.

**Supplementary Information:**

The online version contains supplementary material available at 10.1007/s00442-023-05411-z.

## Introduction

How natural selection shapes the seasonal timing of biological events (phenology) is an important question in evolutionary ecology (Forrest and Miller-Rushing 2010). To date, empirical and theoretical research on phenological adaptation has largely focused on single events (e.g., plant flowering times: Fitter and Fitter 2002; King and Roughgarden 1983; bird migration arrivals: Jonzén et al. 2007; Møller et al. 2008). However, it is important we consider how reproductive success depends on multiple sequential events and developmental processes within an organism’s annual life cycle. For example, wintering ground factors can affect migratory animal condition with knock-on effects to subsequent reproductive timing and success by interacting with breeding ground factors (Bêty et al. 2003). Predicting how organisms adapt to environmental seasonality, therefore, requires us to account for the interdependence of phenological events and cascades of fitness effects acting throughout the annual cycle (McNamara and Houston 2008).

A group of organisms where the interlink of sequential events is likely important in determining reproductive success are the annual social insects, for example many species of bees and wasps (within the genera *Bombus*, *Vespa*, *Polistes*, *Allodape,* and facultatively in *Halictidae*). A typical temperate annual cycle is characterized by four main stages: (i) queen mates and hibernates over winter; (ii) queen emerges, locates a nest site, and forages for food to initiate a colony and start laying eggs; (iii) eggs destined to be workers are reared which forego reproduction to rear more workers; (iv) a switch occurs where eggs laid are reared to be gynes and drones (new queens and males; together termed ‘sexuals’) that are typically larger in body size than workers. Unlike avian or mammalian systems that have one discrete clutch, in social insects, the queen lays consecutive cohorts of offspring (broods), resulting in multiple overlapping generations during stages iii–iv. As foragers, daughter workers increase the amount of resources a colony can collect, which when reinvested into production of new workers can accelerate colony growth via a positive feedback cycle (Heinrich 1979). When the queen starts laying eggs destined to be sexual progeny (stage iii–iv transition; henceforth termed ‘switch point’, cf. Duchateau and Velthuis 1988), colony size in turn determines the level of energetic investment available to rear sexuals, which produce the next generation of colonies. Studying how the queen should schedule her egg laying over time to maximize colony fitness is, thus, critical in understanding phenological adaptation in annual social insects.

Previous theory has largely focused on the transition between production of workers (stage iii) to production of sexuals (stage iv). Based on dynamic energy allocation models (reviewed by Perrin and Sibly 1993), Macevicz and Oster (1976) predicted that the optimal switch point achieves a balance between the benefit of continuing colony growth vs. the length of the sexual production phase. Extensions of their model have considered how the optimal switch point can be predicted by environmental or developmental cues (Hovestadt et al. 2018) or affected by competition for floral resources among colonies (Lindh et al. 2018) and highlighted that a gradual transition to rearing sexuals can be optimal in stochastic environments (Mitesser et al. 2007) or under diminishing returns to growth (Poitrineau et al. 2009; Hovestadt et al. 2019).

The problem of how temporal egg-laying patterns can be adaptive beyond balancing production of workers and sexuals is less explored. Mitesser et al. (2006) showed that a colony growth pattern with distinct breaks in activity, whereby the nest is closed and no new larvae are reared, can be beneficial in the primitively eusocial *Halictidae* if the production loss caused by these breaks can be counterbalanced by increased survival of colony members. Some guidance to this question also comes from models studying the relative benefit of either laying single eggs sequentially and directly supplying them with sufficient food for development (mass-provisioning) or laying multiple eggs simultaneously and feeding them gradually (progressive provisioning) in solitary insects (Field 2005; Mitesser et al. 2017).

We posit that understanding how temporal egg-laying patterns can be optimal in annual social insects requires us to consider their influence on resource sharing across overlapping generations of reared individuals. Frequent egg laying by the queen and larvae being fed over a large portion of their development are characteristic traits of common bumblebee and wasp species which consequently rear multiple developing generations simultaneously. This aspect is, however, sparsely treated in previous theory which either assumes resources collected by the colony is continually transformed to biomass of worker or sexuals (e.g., Macevicz and Oster 1976; Hovestadt et al. 2019), or considers situations where single larvae or single cohorts of even-aged larvae are provisioned at any one time (e.g., Field 2005; Mitesser et al. 2006, 2017). We argue further that theory should consider how body size of emerging adults in addition to optimal number of eggs laid per generation can reflect constraints on colony food income set by the number of active foragers and resource availability in the landscape. Indeed, across annual social insect species, body size can vary greatly between workers and sexuals as well as within these castes (Goulson 2003; Miyano 1983; Richards and Packer 1996). For instance, within bumblebee species, worker sizes can vary over the season (e.g., Knee and Medler 1965; Shpigler et al. 2013), and between species, average body size correlates negatively with colony size (number of workers; Cueva del Castillo et al. 2015), suggesting an inherent number-size trade-off.

Here we address how between-generation competition can influence natural selection on temporal egg-laying patterns in annual social insects. We do this by analyzing a model where the optimal egg-laying rate depends on number-size trade-offs, dynamically changing resource availability, and age-dependent energetic demand of co-existing larval generations. We analyzed our model in three steps, by first predicting optimal egg-laying schedules in a simplified case where necessary resources for larval development are supplied at the egg stage by mass provisioning such that larval generations do not compete for food. Second, we studied the effects of intergenerational competition occurring under progressive provisioning of larvae for optimal egg-laying schedules under parameter settings representing a common and widespread bumblebee species (*Bombus terrestris*). Third, we investigated how the optimal egg-laying schedules are affected by environmental and life-history variables.

## Model and methods

### Model overview

By tracking development and growth of consecutive generations of workers and sexuals, our model: (a) describes how different egg-laying schedules influence the balance between colony energy income and energetic demands of growing larvae; (b) determines an egg-laying strategy that maximizes reproductive output of the founder queen, which we use as the definition of the colony’s optimal reproductive strategy. To help inform our model assumptions and parameters, we used consensus knowledge of development of bumblebee spp. (e.g., Duchateau and Velthuis 1988; Gill et al. 2012; Shykoff and Müller 1995). Our model captures the relationship between individual body size and quality by considering: (i) a positive association between worker body size and foraging efficiency (Goulson et al. 2002; Spaethe and Weidenmüller 2002); (ii) that larger body size of sexuals is beneficial (e.g., improves queen overwintering survival (Owen 1988); increases success rates in male–male competition for mating opportunities (Amin et al. 2012)). While a gradual transition to investment into reproduction (production of sexuals) can be optimal in colony growth models (e.g., Poitrineau et al. 2009), we here assume that this transition occurs in a single time step (bang–bang control) to reduce model complexity.

### Colony demographic dynamics

**Fig. 1:**
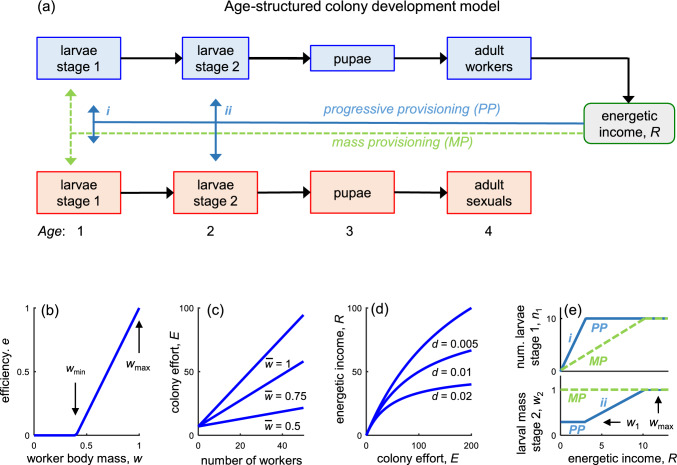
Model overview. **a** Individuals pass through four demographic stages with the emergence of adult workers increasing the colony energetic income, *R*. Under mass provisioning (green arrows), the energy is allocated to larval stage 1. Under progressive provisioning (blue arrows), the energy is first (*i*) allocated to larval stage 1 and second (*ii*) to larval stage 2. **b** The efficiency *e* of an individual worker increases with its body mass above a threshold *w*_min_ until the maximum, *w*_max_, is reached. **c** The total colony effort *E* increases with the number of workers and their body mass (three levels of average body mass $\overline{w }$ are shown). **d** Colony energetic income increases at a decelerating rate with colony effort (three levels of the growth constraint parameter *d* are shown). **e** Number of stage 1 larvae, *n*_1_, and body mass of stage 2 larvae, *w*_2_, as a function of colony energetic income in an examplary situation where these develop from ten eggs laid in the current time step and ten successfully reared stage 1 larvae from the preceeding time step, respectively. Under mass provisioning (MP), the number of stage 1 larvae increases linearly with *R* until all ten individuals are fully provisioned and obtain the maximum body mass, *w*_max_, whereas the second-stage larvae are assumed not to grow. Under progressive provisioning (PP), the number of stage 1 larvae first (*i*) increases with *R* until all ten have obtained their initial body mass *w*_1_. The remaining energy is thereafter (*ii*) allocated to body mass growth of the stage 2 larvae until these have obtained the maximum body mass, *w*_max_

Our model (Fig. 1) is designed as a discrete-time age-structured population model (Caswell 2001; Cresswell 2017) which further describes mean body mass of developing individuals. We let ${n}_{a,t}$ and ${m}_{a,t}$ represent the number of workers and sexuals, respectively, in a generation of age class *a* at time period *t*. The mean body mass of individuals within a generation is represented by ${w}_{a,t}$ for workers and ${v}_{a,t}$ for sexuals.

In *B. terrestris*, worker larvae have been estimated to go into pupation 13 days after they were laid as an egg (Shykoff and Müller 1995) and emerge as adults 9 days later (Duchateau and Velthuis 1988). For model practicality, we considered a 3-week (21 day) developmental stage for workers and assumed that each time step *t* in the model corresponds to 1 week (Fig. 1a). We then categorize week 1 (*a* = 1) as a first larval stage, week 2 (*a* = 2) as a second larval stage, and week 3 (*a* = 3) as the pupal stage. From week 4 and onwards ($a\ge 4)$, an individual is considered as an adult. The lengths of these development stages were also assumed for sexuals (gynes and drones). While development time for gynes and drones is longer than for workers in most social insects (to different degrees), this assumption of sexual development time does not affect the model behavior because reared sexuals do not contribute to colony growth.

For workers, the youngest age class (first larval stage) on week *t* is assumed to develop from the eggs laid that week according to:

1 $${n}_{1,\ {t}}={c}_{t},$$

where ${c}_{t}$ represents the weekly egg-laying rate. Aging and survival to the next week are modeled as:

2 $${n}_{a+1,t+1}={{s}_{a}n}_{a,t},$$

where *s*_*a*_ is the weekly survival rate at age *a*. Larvae and pupae are considered to have a low mortality rate (e.g., Duchateau and Velthuis 1988) relative to adult workers that face risks performing tasks such as foraging (e.g., Gill et al. 2012). For simplicity, we assume no mortality for larvae and pupae (*s*_*a*_ = 1 for *a* = 1, 2 and 3) whereas survival of adult workers, denoted by *S*, may take values below one (*s*_*a*_ = *S* for $a\ge 4$).

We assume that the colony reaches the switch point and starts rearing sexuals at week *t*_*S*_ and that the queen stops laying eggs in week *T*. Dynamics of the number of sexuals is modeled with the same equations as workers but with *n* replaced with *m* in Eqs. 1–2. We assume no mortality of juvenile stages of sexuals which is similar to workers and, following Mitesser et al. (2006), we further assume no mortality of sexual adults during the social phase given the longer life span of adult females (around a year) compared to workers (few weeks).

### Provisioning of energy for colony growth

Following Macevicz and Oster (1976), we treat workers and the founder queen as a collective workforce that rear new adults by collecting nectar and pollen as food provision (energy) without discriminating between the range of specific behaviors that constitute this colony functional role. We assume that the contribution of a generation of workers to collectively work increases linearly with mean adult body mass, ${w}_{a,t}$, and that there is a minimum mean body mass ${w}_{\mathrm{min}}$ below which workers are too small to contribute. We also assume that a worker generation has a maximum mean body mass ${w}_{\mathrm{max}}$, and express the relative efficiency of a generation of workers (Fig. 1b) as a factor between 0 and 1 calculated as:

3 $${e}_{a,t}\left({w}_{a,t}\right)=\mathrm{max}\left(0, \frac{{{w}_{\mathrm{a},\mathrm{t}}-w}_{\mathrm{min}}}{{{w}_{\mathrm{max}}-w}_{\mathrm{min}}}\right),$$

where the denominator acts as a normalizer.

The total amount of work afforded by the colony for producing new workers and sexuals (Fig. 1c) is then considered as:

4 $${E}_{t}= {{r}_{q} +r}_{w}\sum_{a=4,..,N}{n}_{a,t}{e}_{a,t}({w}_{a,t}),$$

where ${r}_{q}$ and ${r}_{w}$ represent the contributions by the founder queen and a full-sized worker, respectively.

Finally, we assume that the energy income to the colony (denoted by *R*_*t*_) increases at a decelerating rate with colony effort (Fig. 1d), corresponding to a law of diminishing returns (Macevicz and Oster 1976; Poitrineau et al. 2009). We believe this consideration to be realistic given likely internal constraints to colony growth, e.g., that biomass production increases at a slower rate than maintenance costs with colony size (Hou et al. 2010), and increasing intra-colony competition for floral resources causes workers to forage further away from the nest (Dramstad 1996). Phenomenologically capturing processes leading to decelerating growth, we assume:

5 $${R}_{t}= \frac{{E}_{t} }{{1+dE}_{t}},$$

where *d* is a factor representing the degree of growth constraints.

### Regulation of larval growth under progressive provisioning

Progressive provisioning of larvae is represented as feeding both first- and second-stage larvae (Fig. 1a, e). For workers, we assume that production of first-stage larvae requires a fixed energetic cost and that these achieve a fixed body mass ${w}_{1}$ after the first week. The body mass of second-stage larvae is assumed to depend on how much energy is invested into this larval stage. Specifically:

6 $${w}_{2,t+1}={w}_{1}+{g}_{t},$$

where *g*_*t*_ represents the weekly average investment in second-stage larvae. We then consider that the energy available, ${R}_{t}$, is transformed directly into larval biomass and following the approach by Beekman et al. (1998), we measure energy income and body mass in the same units. In addition, we assume these units are chosen so that the maximum mean body mass of a worker generation *w*_max_ = 1. We then assume the cost of producing a generation of first-stage larvae in week *t* amounts to ${w}_{1}{n}_{1,t}$. In case the available energy is not sufficient to develop all first-stage larvae from the ${c}_{t}$ eggs laid (i.e., when ${{R}_{t}\le w}_{1}{c}_{t}$), we set ${n}_{1,t}$ = ${{R}_{t}/w}_{1}$ and ${g}_{t}=0$ (Fig. 1e, top). Otherwise (i.e., when ${{R}_{t}>w}_{1}{c}_{t}$), the remaining energy is next used to grow the second-stage larvae (if present). We then assume second-stage larvae have a maximal growth rate ${g}_{\mathrm{max}}= {w}_{\mathrm{max}}-{w}_{1}$ resulting in mean body mass never exceeding ${w}_{\mathrm{max}}$ (Fig. 1e, bottom). It follows that also the amount of energy required to grow a single worker to this size (*g*_max_ + *w*_1_) = 1. Growth of the second-stage larvae is, hence, modeled as:

7 $${g}_{t}=\mathrm{min}\left({g}_{\mathrm{max}},\frac{{R}_{t}- {w}_{1}{n}_{1,t}}{{n}_{2,t}}\right).$$

If any energy remains after the second-stage larvae have reached ${w}_{\mathrm{max}}$ or if no second-stage larvae are present, for the sake of our model, we assume any surplus energy (i.e., ${{R}_{t}-w}_{1}{n}_{1,t}-{{g}_{\mathrm{max}}n}_{2,t}$) is generally wasted given bumblebees store relatively little food (Goulson 2003).

Individual growth of sexuals is modeled in the same way as workers but with $n,w,$ and ${g}_{\mathrm{max}}$ replaced with $m,v,$ and ${h}_{\mathrm{max}},$ respectively (Eqs. 6–7 and corresponding expressions). However, note that energy provisioning (Eqs. 3–5) depends exclusively on workers, as new sexuals are not considered to forage for the colony.

### Mass provisioning of larvae

Mass provisioning, where all energy required for larval development is supplied in connection to egg laying (Field 2005), is represented by energy being supplied to first-stage larvae only (Fig. 1a, e). We implement this by letting first-stage larvae obtain sufficient energy to attain maximal body mass, so that ${w}_{1}={w}_{\mathrm{max}}$ which implies ${g}_{\mathrm{max}}=0$, and hence no growth of second-stage larvae (Eq. 7, Fig. 1e). We further interpret ${w}_{1}$ as the energy supplied to the larvae for subsequent growth rather than its actual body mass. Should the available energy not be sufficient to develop all larvae from the ${c}_{t}$ eggs laid (i.e., when ${{R}_{t}<w}_{\mathrm{max}}{c}_{t}$), we set ${n}_{1,t}$ = ${{R}_{t}/w}_{\mathrm{max}}$, leading to a reduction in the number of adults produced.

### Reproductive success and optimization of the egg-laying strategy

We assume that the body mass of gynes and drones reflect their quality with a linear relationship between the two, calculated as a factor ${e}_{a,t}$ between 0 and 1 using Eq. 3 but with ${w}_{\mathrm{min}}$ and ${w}_{\mathrm{max}}$ replaced with ${v}_{\mathrm{min}}$ and ${v}_{\mathrm{max}}$. As a fitness proxy, we then calculate the reproductive success *F* of a colony based on total production of sexuals in terms of number and quality at emergence (cf. Eq. 4):

8 $$F= \sum_{\forall t}{m}_{4,t}{e}_{4,t}\left({v}_{4,t}\right).$$

We define the optimal egg-laying schedule as the set of clutch sizes ${c}_{t}$ for *t* = 1,…,*T* and the switch point *t*_S_ which maximizes the reproductive success *F* (Eq. 8). This optimum may represent either the outcome of long-term adaptation by natural selection in a stable environment (Kozłowski 1993) or the optimal plastic response to a certain environmental condition assuming the (super-)organism is able to anticipate this condition (perfect knowledge) and further adapt its behavior at no fitness cost (no limits of plasticity) (Parker and Maynard Smith 1990). The optimal egg-laying schedules were calculated numerically using the Nelder–Mead method (fminsearch) in Matlab (code available in Supplementary Information Appendix S3). The algorithm searches for local optima. However, we found it was insensitive to initial conditions and that the solutions consistently resulted in colony energy demand and supply being balanced over the colony life cycle (see below), indicating that the identified optima are global and biologically sensible.

### Parameter values

As an empirical reference scenario, we model the *B. terrestris* life history using the following parameter values: $T=10, S=0.8,d=0.01,{ r}_{\mathrm{q}} =7,{ r}_{\mathrm{w}} =1.75,{ w}_{1} =0.3, {g}_{\mathrm{max}}= 0.7, {w}_{\mathrm{min}}=0.4,{w}_{\mathrm{max}}=1, { v}_{1}=0.6,{h}_{\mathrm{max}}= 1.4, {v}_{\mathrm{min}}=0.6,{v}_{\mathrm{max}}=2$. For derivations and motivation, see Supplementary Information Appendix S1. Throughout, we assume that parameters controlling body size growth of sexuals scale with the corresponding worker parameters by ${v}_{\mathrm{max}}$ such that ${v}_{1}={v}_{\mathrm{max}}{w}_{1},{h}_{\mathrm{max}}= {v}_{\mathrm{max}}{g}_{\mathrm{max}}, {v}_{\mathrm{min}}={v}_{\mathrm{max}}{w}_{\mathrm{min}}.$

## Results

### Colony development in a simplified scenario

**Fig. 2:**
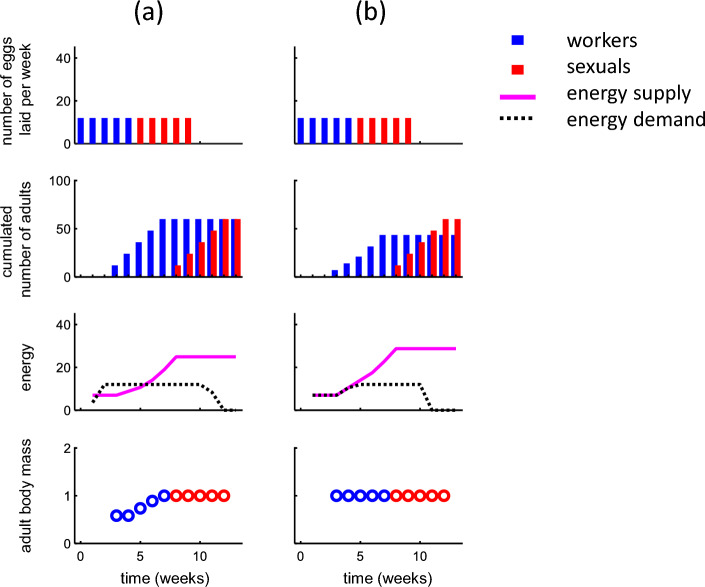
Illustration of colony dynamics in a simplified scenario under progressive provisioning (**a**) and mass provisioning (**b**). The queen is assumed to lay eggs at a constant rate (first row) causing the cumulated number of adults (second row) to increase. Note the 3-week developmental lag between when eggs are laid and adult emergence. In early colony stages, energy demand exceeds supply (third row). This energy deficit leads to smaller adults (last row) under progressive provisioning and to fewer adults being produced under mass provisioning

To illustrate basic model behavior, we start by considering a case with identical maximum body size for workers and sexuals (${v}_{\mathrm{max}}=1$), no mortality (*S* = 1), no growth constraints (*d* = 0), and low productivity (${r}_{w}=0.5$) but remaining parameters as in the *B. terrestris* reference scenario (see Parameter values above). We then consider the simple (but non-optimal) strategy of laying a constant number of eggs per week (*c*_*﻿t*_ = 12 for all *t*) and switch between producing workers and sexuals in the middle of the colony cycle (*t*_s_ = 6, Fig. 2). Under progressive provisioning (Fig. 2a), the constant laying rate leads to a linearly increasing number of worker adults followed by a linearly increasing number of sexuals (Fig. 2a, second row). In the first weeks, the energy supplied (by the queen) is lower than the (constant) demand (Fig. 2a, third row) resulting in the first generation of workers obtaining below-maximum body mass (Fig. 2a bottom row). Energy supply increases as the colony grows, and when it exceeds demand (from week 5), adults obtain the maximal body size. Under mass provisioning (Fig. 2b), the same laying strategy results in a qualitatively similar growth pattern of number of adults per week, except that the relative energy shortage in the first weeks is reflected in reduced number of adult workers being produced rather than in reduced body mass.

### Optimal egg-laying schedules under mass provisioning

**Fig. 3:**
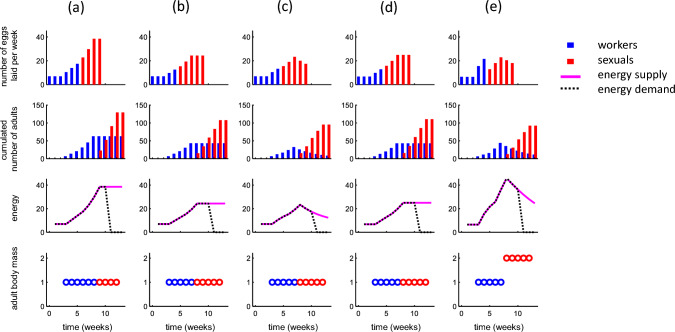
Optimal egg-laying schedules under mass provisioning, and hence in the absence of competition among larval generations. **a** The optimal strategy under the same parameter settings as in Fig. 2b. The following columns of panels show the effect of reduced worker productivity (**b** *r*_w_ = 0.4), reduced survival (**c** *S* = 0.8), growth constraints (**d** *d* = 0.005). The last column (**e**) shows the optimal strategy for the *B. terrestris* reference scenario but assuming mass provisioning

Figure 3a shows the optimal egg-laying schedule under mass provisioning, and hence without intergenerational competition with the same simplified parameter setting as in Fig. 2. The queen first lays a constant number of eggs per week (until week 3), then gradually increases her laying rate until a plateau with a high constant rate is reached at the end of the season (weeks 9–10). This egg-laying pattern generates an accelerating increase of the cumulated number of worker adults (Fig. 3a, second row) and thereby of the energy supply (Fig. 3a, third row). In contrast to the non-optimized egg-laying schedules in Fig. 2, the energy supply curve here matches the energy demand curve (Fig. 3a, third row) resulting in body size being maximized (Fig. 3a, bottom row). Note finally, how the length of the developmental stage (4 weeks) and the optimized switch point (first sexual eggs laid week 6) influence the egg-laying schedule. Egg-laying rates are constant when the queen is the sole energy provider, and start to increase when the first adult workers emerge (week 4) becoming constant after the last worker eggs (laid week 5) have developed into adults (week 9).

Figure 3b–d shows how the optimal egg-laying strategy and the associated colony growth pattern is affected by model parameters. In all cases, the optimal strategies result in matching supply with demand (cf. Figure 2). Reduced worker productivity results in a more slowly accelerating colony growth and an earlier switch (Fig. 3b). Worker mortality causes slower increase in the number of workers, an earlier switch, and a decline in worker numbers in later colony stages (Fig. 3c). Note in this case, however, that the number of adults continues to increase after the switch point to reach a peak at 3 weeks into the sexual egg-laying phase because of the temporal delay between egg laying and emergence of adults. This also implies that energy supply and optimal egg-laying rate peak after the switch. Growth constraints slow down colony growth, and leads to an earlier switch (Fig. 3d). The colony growth patterns generated by the optimal strategy are broadly similar to those described by the Macevicz and Oster (1976) model (in particular to extensions that account for larval development time, e.g., Mitesser et al 2006; Hovestadt et al. 2018) and respond similarly to parameter variation including regarding optimal switch times (see Supplementary Information Appendix S2 for details).

The optimal strategy for parameters of the *B. terrestris* reference scenario but under mass provisioning (${w}_{1}={w}_{\mathrm{max}}$, ${g}_{\mathrm{max}}=0$) is shown in Fig. 3e. The larger body size of sexuals assumed in this scenario (${v}_{\mathrm{max}}=2$) results in a relatively low late-season egg-laying rate due to the increasing per capita energy demand (cf. Fig. 3a–d). Otherwise, colony and energy dynamics are similar to those in Fig. 3b where worker mortality implies that number of workers, energy balance, and sexual eggs peaks 3 weeks into the phase of sexual egg laying.

### Effects of intergenerational competition under progressive provisioning

**Fig. 4:**
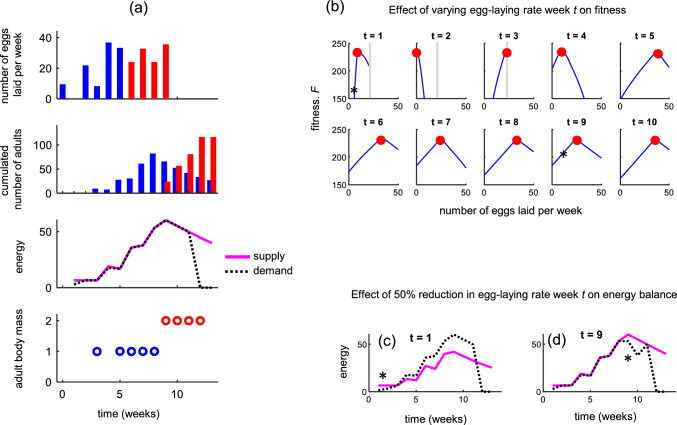
The optimal egg-laying strategy under progressive provisioning and competition among larval generations in the *B. terrestris* reference scenario. **a** Egg-laying schedule and colony development represented as in Figs. 2–3. **b** Sensitivity of the optimal egg-laying strategy. Each panel in [**b**] shows how colony reproductive output is affected by unilateral changes in the number of eggs laid in any given week *t*, while the number of eggs laid in all other weeks remain at their optimal values (denoted by red, filled circles). Grey vertical lines represent the maximal number of eggs that can possibly be laid each week (not shown if above 50) given the energetic income *R*_*t*_/*w*_1_. The effect on energy balance of unilaterally halving the number of eggs early (*t* = 1) and late (*t* = 9) is shown in **c** and **d**, respectively

The optimal egg-laying schedule for parameters representing *B. terrestris*, with intergenerational competition occurring due to progressive provisioning, is shown in Fig. 4a. Initially, there are two temporally separated egg-laying peaks (week 1 and 3 in Fig. 4a, top row) with eggs thereafter laid at a relatively high albeit uneven rate (week 5 onwards). Comparing the optimal strategies under mass provisioning (Fig. 3e) and progressive provisioning (Fig. 4a) holding all else equal shows that competition among larval generations for resources causes a larger variation in egg-laying rate between consecutive generations, especially early in the colony cycle. Under progressive provisioning, the optimal switch point is also later and fitness higher (Fig. 3e vs. Fig. 4a with 116 and 92 reared sexuals, respectively).

We studied the sensitivity of this optimal strategy by systematically varying the number of eggs laid in a specific week, one at a time, while keeping the number of eggs laid in all other weeks at their optimal values (Fig. 4b). This revealed the optimal egg-laying strategy to be more sensitive to changes in the number of individuals in the early as opposed to later generations (i.e., blue lines in Fig. 4b have steeper slopes) due to the stronger knock-on effect that these changes have on future colony growth (Fig. 4c–d). In particular, by decreasing the number of eggs laid in the first week or increasing them in the second considerably decreased final reproductive output. Decreasing the number of workers reared in the first generation constrains the amount of energy that can be provisioned for body mass growth of the third generation which in turn constrains subsequent colony growth. Increasing the number of individuals of the second generation (from zero) instead compromises body mass in the first, and thereby indirectly the body mass in the third. These considerations explain why producing eggs during the first and third week with no eggs in between is optimal. Since egg laying in the latter stages has a weaker knock-on effect on future colony growth (Fig. 4b–d), the relative benefit of holding-back egg laying to reduce energy conflicts among consecutive generations diminishes. This, in turn, explains why variation in egg-laying rates becomes less pronounced over time.

### Dependence of the optimal strategy on environmental and life-history-related parameters

**Fig. 5:**
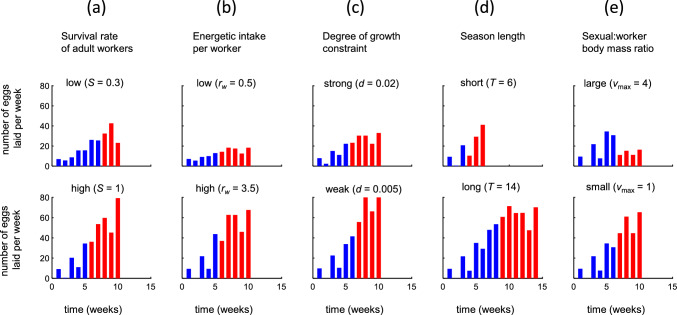
Effect of environmental and life-history parameters on optimal egg-laying schedules under progressive provisioning. Each panel shows the effect of changing one specific parameter (top of each column) at the time with the remaining parameters set according to the *B. terrestris* reference scenario (shown Fig. 4a). Notation and symbols are otherwise as in Fig. 4a, top row

We unilaterally increased or decreased one parameter at a time (Fig. 5) in relation to the *B. terrestris* reference scenario (Fig. 4a). Overall egg-laying rates are negatively affected by worker mortality (Fig. 5a), reduced energy intake (Fig. 5b), growth constraints (Fig. 5c), and shorter seasons (Fig. 5d) similarly to the mass-provisioning scenario above (Fig. 3) and responses of optimal switch points to variation in these parameters align with previous theory (see Supplementary Information Appendix S2 for details).

In most settings (Fig. 5), the optimal strategy exhibits strong between-week variation in egg-laying rate early in the colony cycle, which is similar to the reference scenario (Fig. 4). However, under low survival (Fig. 5a, top row) and low worker productivity (Fig. 5b, top row), it is optimal to lay eggs in the second week and laying rates of worker-destined eggs show a steadier and more gradual increase. In the *B. terrestris* reference scenario (Fig. 4a), laying only a few or even zero eggs in certain weeks during the initial stages reduces competition between young and old larvae. When survival is decreased, however, workers born early will die off sooner, and therefore contribute less energy to growth of subsequent generations (Fig. 5a). Thereby reducing the number of individuals of a younger generation to improve body mass, growth of an older one becomes less beneficial. Similarly, the value of older individuals for future colony growth is reduced when energy intake is decreased (Fig. 5ab) because comparatively they will not be able to boost the growth of subsequent generations to the same extent.

At later stages of colony development, egg-laying rates show less between-week variation and are relatively constant in several settings (Fig. 5), which is again similar to the reference scenario (Fig. 4a). This is partly due to growth constraints (*d*), which implies that late-season colony energy income is relatively constant even though the number of adult workers first increases after the switch point (due to maturation delay) and then declines (due to mortality). As a corollary, we find more pronounced between-week variation in latter colony stages under weak growth constraints (small *d*) (Fig. 5d) and when colony growth is limited by a short season (Fig. 5e) rather than by negative density dependence. Finally, we note that egg-laying rates after the switch point decreases with the body size of sexuals relative to workers (Fig. 5e) but that this factor does not affect the temporal pattern of laying of worker-destined eggs (cf. Figure 3e).

## Discussion

### Optimal egg-laying schedules: model predictions and empirical patterns

Our analysis sheds light on how egg-laying schedules can be optimized for annual social insects that rear overlapping generations of developing larvae. We considered the situation where larvae are fed repeatedly during their development (progressive provisioning) implying that co-existing larval generations compete for food. We compared it to a situation where one larval generation is supplied at the time (mass-provisioning) such that no intergenerational competition occurs. In the non-competitive situation, the optimal strategy is to gradually adjust the egg-laying rate in proportion to colony energetic income, resulting in a smooth and continuous colony growth pattern qualitatively similar to the classic model by Macevicz and Oster (1976). In contrast, when larval generations compete, optimal egg-laying schedules appear irregular with considerable variation in the number of eggs laid between consecutive generations.

We show that between-generation variation in egg-laying rates can be adaptive by minimizing competition for resources between young and older larvae. Focusing on the early part of the colony cycle, our model also shows that pausing egg-laying can be optimal for this same reason, providing an adaptive explanation to why annual social insect species have been reported to produce discrete broods separated by interim periods rather than a continuous and regular pattern of laying. Indeed, in *B. terrestris* (Duchateau and Velthuis 1988), *B. hypocrita*, *B. lucorum,* and *B. ignita* (Asada and Masato 2000; Li et al. 2008), the queen has been observed to initiate a colony by producing two temporally separated broods—a smaller clutch followed by larger. Interestingly, our model also predicts this small-followed-by-large egg-laying pattern (Fig. 4) in our baseline scenario, providing support for the validity of our model.

Our model predicts that temporally separated broods are not adaptive when worker survival or energy intake decreases and that in such cases, eggs should instead be laid continuously but at a gradually increasing rate (Fig. 5a, b). Given these findings, it is interesting that the queen in the paper wasp *Polistes chinensis antennalis* appears to initially lay eggs continuously but at a low rate and then increases egg-laying rate once the first workers have emerged (Miyano 1983). This might be an adaptation to low worker survival, as we predict (Fig. 5a), given that worker mortality has been reported to be high in the *Polistes* genus (Greene 1984; but see: Miyano 1983).

We predict a transition from high to relatively low variation in between-generation egg-laying rates over time as the colony develops (Figs. 4, 5). Furthermore, in the baseline scenario, optimal laying rates in later colony stages are relatively constant due to colony growth constraints. These predictions are compatible with observations of constant egg-laying rates in the latter part of the colony cycle (so called linear phase in *B. terrestris*; Duchateau and Velthuis 1988). However, optimal egg-laying rates in later colony stages can decrease near the end of the colony cycle with low worker survival or large body size of sexuals relative to workers. Given that sexual:worker body size ratios are known to vary across annual social insects, our model suggests that a linear phase is not always an adaptive feature. We further predict that there is no period of relatively constant egg laying under a short season where instead the optimal pattern consists of two initial worker broods followed by an increasing number of sexual eggs (Fig. 5d). Corroborating this, Arctic bumblebee species such as *B. polaris* or *B. lapponicus* are thought to lay one or two clutches of worker eggs before switching to production of sexuals (Richards 1973; Martinet et al. 2022).

To test our predictions, future studies could investigate: (i) patterns of local adaptation in egg-laying strategies along geographic and environmental gradients within or among species, or (ii) plastic responses of egg-laying rates to different social and external environmental conditions, such as variation in paternal lines, food availability, and survival rates. To test our assumptions of how cross-generation interactions and energy balance impacts colony performance, future experimental studies could also investigate the effects of manipulating egg rate or worker number on precise measures of energy consumption, colony growth, and reproductive output.

### Comparison with previous theory and model robustness

While our analysis highlights how competition between different larval generations can affect optimal egg-laying schedules, the colony growth patterns and switch points of optimal strategies predicted by our model and the Macevicz and Oster (1976) model respond similarly to variation in model parameters. Our model also predicts an overall pattern of growth followed by a decline in worker number matching previous theory (e.g., Macevicz and Oster 1976; Hovestadt et al. 2019) as well as empirical observations (Crone and Williams 2016; Malfi et al. 2022). Further, our predictions regarding egg-laying patterns are at least partially consistent with the study by Mitesser et al. (2017) who showed that solitary insects with overlapping generations, e.g., multivoltine species, obtain a higher geometric population growth rate when the queen lays temporally separated broods and feed these progressively rather than laying one mass-provisioned egg after another. Here, we similarly find that laying temporally separated broods under progressive provisioning results in a faster colony growth rate, and thus higher fitness than laying at a more even rate under mass provisioning (Figs. 3e, 4a). Mitesser et al. (2017) suggested that the former strategy would be adaptive for social insects during the worker-dominated colony growth phase whereas mass provisioning with serial laying of single eggs would be adaptive during the reproductive phase. Here we similarly predict that egg-laying rates should be less variable at later colony stages, but importantly we show that this reduced variability can be adaptive for social insects that consistently use progressive provisioning throughout their colony cycle.

We here considered a relatively coarse time scale (approximately weekly) compared to previous related models. This enabled us to consider competition among a minimum number (2) of larval age groups, while still being a plausible frequency for monitoring experimental colonies (e. g., Gill et al. 2012; Watrobska et al. 2021) making the model amenable to empirical tests. While we expect our main results to hold on a finer time resolution, studying this requires stipulating additional rules for how multiple larval stages develop and interact, which in turn may influence optimal strategies. The assumption that colonies produce workers and sexuals in separate periods (bang-bang control) is another simplification in our model. Optimal reproductive allocation schedules can contain periods of mixed allocation (e.g., Poitrineau 2009) and in general be diverse in shape (Engen and Saether 1994; Mitesser et al. 2006; Johansson et al. 2018). Since we predict that variation in egg-laying rate between consecutive clutches is higher in the worker-dominated early phase than in the later phase of sexual production, a graded transition to producing sexuals could possibly result in a more gradual decrease of this variation over the colony cycle.

By taking an optimality approach, our model assumes full behavioral flexibility and perfect knowledge which we should note may not be wholly realistic. For example, behavioral responses in egg-laying rates could be restricted by queen physiological limits in egg laying rate per day (Beekman et al. 1998), although in many perennial social insects, queens are capable of laying 100–1000 s of eggs per day (Bodenheimer 1936) showing that such strategies have the potential to evolve. Egg-laying strategies might also be influenced by inter-annual variation in seasonal conditions and unpredictable resource availability. In great tits (*Parus major*), for example, small clutch sizes may improve offspring food provisioning in bad years (Boyce and Perrins 1987). Annual social insects exhibit a range of behaviors to deal with energy shortfalls, such as larval ejection to reduce energetic demand (Roger et al. 2017), adaptively recruit a higher proportion of the workforce as foragers (Cnaani and Hefetz 1994), long-term food storage (Heinrich 1979) or prolonged development times (Sutcliffe and Plowright 1990). By assuming that rearing of young larvae is prioritized over rearing of older, resource shortage in our model results in reduced body sizes, as observed in bumblebees (Schmid-Hempel and Schmid-Hempel 1998). In our model, we did not consider storage of excess food since this is not very prominent in bumblebees compared to, for example, honey bees (Goulson 2003). The exact assumptions of the behavioral response to resource variability, however, do not appear critical for the present analysis. Since we predict that colonies using optimal egg-laying strategies to achieve a supply–demand balance, they would remain optimal even if the cost of deviating from perfect energetic balance would take other forms, e.g., delayed investments due to storage or wasted production due to larval ejection. By analyzing effects of stochastic environments on our predictions, future research could shed light on selection of different strategies to buffer environmental uncertainty, including more cautious egg-laying strategies than predicted here. Other interesting extensions include considering that smaller workers have longer life expectancies to offset lower foraging efficiency (Kerr et al. 2019) and/or that worker kin selective benefits influence laying rates or timing of the switch point (Avila et al. 2019) whether via direct fitness benefits through worker laid males or indirect fitness benefits through queen coercion (Gill and Hammond 2011).

### Final remarks

Our model provides a theoretical framework to understand how egg-laying strategies of annual social insects can be adaptive under different environmental conditions and how this, in turn, can be linked to energy supply–demand dynamics and number-size trade-offs. Understanding which life-history strategies benefit from or become disadvantaged by global environmental change, and how evolution may play out in different future scenarios will be important in protecting and managing wild populations in the long term (Ferriere et al. 2004; Gill et al. 2016). In this context, a general theory predicting how strategies evolve from first principles, like we present here, can be useful for testing how scenarios of environmental perturbations affect colony demography and reproductive success and support conservation efforts targeting annual social insects.

## Supplementary Information

Below is the link to the electronic supplementary material.

Supplementary file1 (PDF 918 KB)

## Data Availability

No biological data were collected for this theoretical study**.**

## Code availability

The Matlab code is provided in Appendix S3 in the Supplementary Information.
