# Supplementary material for: How competition between overlapping generations can influence optimal egg-laying strategies in annual social insects
Source: Oecologia. 2023 Jul 10;202(3):535–47. doi: 10.1007/s00442-023-05411-z (PMC10386978; doi:10.1007/s00442-023-05411-z)
Supplement: Supplementary file 1 — Supplementary file1 (PDF 918 KB) [file 442_2023_5411_MOESM1_ESM.pdf]

Supplementary Information

How competition between overlapping generations can influence optimal egg-laying strategies in annual social insects

Jacob Johansson[*,1,2] Andres Arce[2,3] & Richard J. Gill[2]

[1] Department of Biology, Lund University, Sölvegatan 37, 22362 Lund, Sweden

[2] Georgina Mace Centre for the Living Planet, Department of Life Sciences, Imperial College London, Silwood Park Campus, Buckhurst Road, SL5 7PY Ascot, Berkshire, UK

[3] School of Engineering, Arts, Science & Technology, University of Suffolk, 11 Ipswich, IP3 0FS, UK.

[*] correspondence: jacob.johansson@biol.lu.se

Appendix S1: Motivation of baseline parameter values

Parameter values of our model are rounded to facilitate interpretations of graphs and calculations. The parameters w_1 and $g_{\max}$ represent the larval demands of energy for growth during the first and second week, respectively (a = 1 and 2). We assume pollen consumption and larval mass undergoes an exponential-like increase with time, in which only a small proportion of the larval weight gain occurs during the first week (Ribeiro et al. 1993; Cnaani and Hefetz 1994). That said, rearing early stage larvae is still not cheap as incubation and temperature regulation are needed (Heinrich 1979) and nectar storage requires frequent replenishment (Sladen 1912). To capture the asynchrony in larval demands between early and late stage larvae by setting $w_1 = 0.3$ and $g_{\max} = 0.3$ equating to late stage larvae (a = 2) requiring more than double the energy for growth to early stage larvae (a = 1). We then set the minimal adult worker body mass $w_{\min} = 0.4$. For simplicity, we then assume energy costs and minimum body sizes of sexuals are proportional to those of workers, but scaled with their maximum body size by assuming $v_1 = w_1 v_{\max}$ and $v_{\min} = w_{\min} v_{\max}$.

Lopez-Vaamonde et al. (2009) estimated the mass of individual gynes and drones to 799 mg and 266 mg respectively, or 3.8 and 1.25 times the mass of a worker (estimated to 210 mg by Holehouse, Hammond, & Bourke [2003]). Considering that a colony typically produces more drones than gynes, our model assumes sexuals on average require twice as much energy than workers and set $v_{\max}$ = 2.

To estimate the contribution of the founder queen (r_q) and the workers (r_w) to the work afforded by the colony, we consider a stylized *B. terrestris* egg-laying pattern where a first generation of workers (eggs laid week 1) help to rear a second generation of workers (eggs laid week 3) . Beekman et al (1998) estimated that the first and second brood consisted of 9.7 and 36.7 workers respectively based on data in Duchateau and Velthuis (1988). Approximating these numbers we assume the queen on her own has the capacity to provide sufficient energy for rearing one generation of 10 full-sized workers, and then together with these is able to rear a second generation of up to 35 full-sized workers.

Because the second larval stage is the most energetically-demanding stage, requiring an amount of energy $g_{\max}$, we require that $r_{\mathrm{q}} = 10g_{\max}$ and $r_{\mathrm{q}}+10r_w = 35g_{\max}$ resulting in the assumption that $r_q = 7$ and $r_w = 1.75$.

The parameter d sets an upper limit to the number of eggs that can be produced (R_t approaches $1/d$ when E_t becomes large [eq. 5]. Duchateau and Velthuis (1988) estimated that colonies during the later continuous rearing phase produced 1.1 and 2.1 egg cells per day, respectively, in two separate experiments, with each egg cell containing on average 8 eggs. This corresponds to a laying rate of between 70 – 140 eggs per week and here we assume a colony can rear a maximum of 100 adult workers per week and thus set d = 0.01.

Adult survival S was set to 0.8 corresponding an average life span of 3.1 weeks (median worker longevity may range from 16 to 76 days; Smeets & Duchateau [2003]). Season length T, here defined as the time span between the laying of the first worker eggs and the last sexual eggs, was set to 10 weeks, corresponding to a queen longevity of 70 days from colony initiation (cf. Lopez-Vaamonde et al. 2009).

References

Cnaani J, Hefetz A (1994) The effect of workers size frequency distribution on colony development in Bombus terrestris. Insectes Soc 41:301–307. https://doi.org/10.1007/BF01242301

Holehouse KA, Hammond RL, Bourke AFG (2003) Non-lethal sampling of DNA from bumble bees for conservation genetics. Insectes Soc 50:277–285. https://doi.org/10.1007/s00040-003-0672-6

Lopez-Vaamonde C, Raine NE, Koning JW, et al (2009) Lifetime reproductive success and longevity of queens in an annual social insect. J Evol Biol 22:983–996. https://doi.org/10.1111/j.1420-9101.2009.01706.x

Ribeiro M, Velthuis H, Duchateau MJHM (1993) Growth in bumblebee larvae: relations between the age of the larvae, their weight and the amount of pollen ingested by them. Proc Exp Appl Entomol NEV Amsterdam 4:121–125

Sladen FWL (1912) The humble bee: its life history and how to domesticate it. MacMillan and Company, London

Smeets P, Duchateau MJ (2003) Longevity of bombus terrestris workers (Hymenoptera: Apidae) in relation to pollen availability, in the absence of foraging. Apidologie 34:333–337. https://doi.org/10.1051/apido:2003026

Appendix S2: Comparison with previous dynamic energy allocation models for annual social insects

In a first section, we here compare the colony growth patterns generated by the optimal egg-laying schedule in our model under mass provisioning with the colony growth pattern described by the dynamic energy allocation model introduced by Macevicz and Oster (1976). Thereafter we compare how the timing of the optimal switch point is affected by model parameters in our model and the Macevicz and Oster (1976) model alongside further studies building thereon.

Colony growth patterns

We focus on colony development in terms of growth of number of workers and sexuals shown in Fig. 3a-d (second row). We will first consider a hypothetical case with unconstrained colony growth and no worker mortality (as in Fig. 3a) and then in turn study the effects of reduced productivity (as in Fig. 3b), worker mortality (as in Fig. 3c) and growth constraints (as in Fig. 3d).

Macevicz and Oster (1976) analysed a model with unconstrained growth defined by:

$$\frac{dW}{dt} = bR(t)u(t)W(t) - \mu W(t) \qquad \text{with } W(0) = W_0, \qquad \text{(eq. S2a)}$$

$$\frac{dQ}{dt} = cR(t)(1 - u(t))W(t) - vQ(t) \qquad \text{with } R(0) = 0, \qquad \text{(eq. S2b)}$$

where, t is time, W is the number of workers, Q is the number of sexuals produced by the colony, $R(t)$ is resource availability, b is a energy conversion factor, μ and v are mortality rates of workers and sexuals, respectively, and $u(t)$ is the fraction ($0 \leq u \leq 1$) of colony resources allocated to production of workers as opposed to production of sexuals.

Macevicz and Oster (1976) also considered a model of constrained colony growth by modifying equation S2a to:

$$\frac{dW}{dt} = b_0R(t)u(t)W(t)(1 - b_1W(t)) - \mu W(t) \text{ with } W(0) = W_0 \qquad \text{(eq. S2c)}$$

where b_0 corresponds to b above and the parameter b_1 reduces the growth rate at high densities, leading to logistic growth of the number of workers (unless the colony switches to produce sexuals or the season ends).

As in the main paper, we assume that resource availability is constant over time by setting $R(t) = R_M$ for all t, that sexual mortality $v = 0$ and that the population use a bang-bang strategy and switches from producing worker to producing sexuals at a time point t_M with $u = 1$ for $0 < t < t_M$ and $u = 0$ for $t_M < t < T$ and. Note that Macevicz and Oster (1976) define the switch point (here t_M) as the time when sexual adults are first produced whereas the switch point (t_S) in our model refers to when the first sexual eggs are laid. Therefore t_S corresponds to t_M plus the length of the developmental stage (3 weeks). For simplicity we set $b = c = b_0 = 1$ and regulate productivity by varying R_M. Note that these settings imply that the model for unconstrained growth (eq. S2a-b) is equivalent to the model for constrained growth (eq. S2c) when $b_1 = 0$. We set the value of the switch points to their optimal values using the analytical results by Macevicz and Oster (1976) for unconstrained growth and using numerical calculation for constrained growth since there is no explicit solution for this case (cf. Lindh et al 2016).

Fig. S1 shows colony development in terms of growth of workers (W) and sexuals (Q) with parameter variation corresponding to Fig. 3a-d calculated from the model above (eqs. S2a-c). We start by focusing on the dynamics of workers. With unconstrained growth (eq. S2a) and no worker mortality (Fig. S1a), the number of workers grows at an accelerating rate until the switch to production of sexual adults (t_M) and remains constant thereafter similar to Fig. 3a. When productivity is reduced (Fig. S1b), the number of workers grow at a slower rate, but follow the same overall pattern, similar to Fig. 3b. The effect of worker mortality (Fig. S1c) is a lower growth rate of workers prior to the switch to production of sexual adults (t_M) and decline in worker numbers thereafter, similar to Fig. 3c. The effect of constrained growth (eq. S3) is a lower growth rate of workers, similar to Fig. 3d.

Concerning production of sexuals, the Macevicz and Oster (1976) model show the same effects of parameter variation on colony fitness as our model. Specifically, the cumulated number of sexuals produced at the end of the season becomes lower due to reduced

productivity (Fig. 3b, Fig. S1b), worker mortality (Fig. 3c, Fig. S1c) and constrained growth (Fig. 3d, Fig. S1d) in both models.

Some differences between the colony growth patterns in Fig. 3 and Fig. S1 occur since our model, in contrast to the one by Macevicz and Oster (1976), accounts for larval development time. For example, in our model the number of sexuals produced by the end of the season is larger than the maximum number of workers (Fig. 3a-c) in cases where these numbers are equal in the Macevicz and Oster model (Fig. S1a-c). This is because the time lag between egg-laying and emergence of adults implies that the number of adults and thereby colony resources continue to grow for a period of time after the first sexual eggs are laid. In the models by Mitesser et al (2006) and Hovestad et al (2019), which also include larval development time, the number of sexuals produced by the end of the season can be larger than the maximum number of workers, similar to here. It can also be noted that the increase in number of workers after the switch to laying of sexual eggs affects the curvatures of the cumulated number of sexual adults as a function of time. For example, when there is no worker mortality, the number of sexuals produced grows linearly in the Macevicz and Oster (1976) model (Fig. S1a,b), whereas it grows at an accelerating rate in our model (e.g. Fig. 3a). A similar effect can be seen in the model by Mitesser et al (2006; their Fig. 1a) where the number of sexuals grow at an accelerating rate in a case where workers are subject to mortality and the Macevicz and Oster (1976) model predicts it should grow at a decelerating rate (Fig. S1c).

Overall, these comparisons show that colony growth patterns predicted by our model under mass-provisioning (Fig. 3) are broadly similar to those described by the Macevicz and Oster (1976) model, if we disregard the coarser, discrete time scale in our model. The inclusion of larval development time in our model affects colony growth patterns in line with results from models that extend the Macevicz and Oster model with this feature (Mitesser et al. 2006, Hovestad et al. 2018).

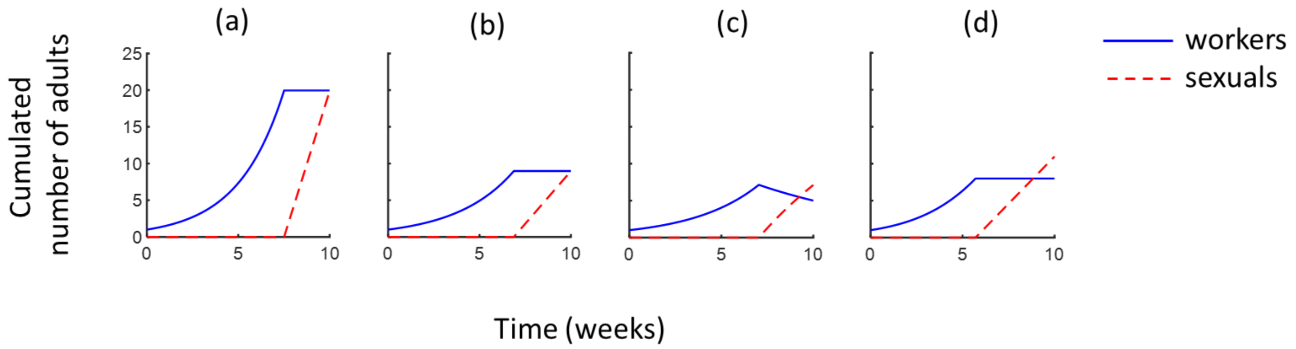

Figure S1. *Colony development as described by the Macevicz and Oster (1976) model for comparison with Fig. 3a-d in the main text. Starting from simplified case with unconstrained colony growth and no worker mortality (a) the simulations show the effects of reduced productivity (b), worker mortality (c) and growth constraints (d). The colony dynamics was calculated using eqs. S2b-c. Parameter values:* $b_0 = c = W_0 = 1$, $R_M = 0.4$ *and* $b_1 = \mu = v = 0$ *except in b)* $R_M = 0.32$, *c)* $\mu = 0.12$ *and d)* $b_1 = 0.025$.

Optimal timing for switching to reproduction

Based on their model and for the case of unconstrained, exponential colony growth (eq. S2a). Macevicz and Oster (1976) calculated that the optimal switch point occurs earlier when worker mortality increases and when worker productivity decreases. In the scenario of mass-provisioning in our model we see these same effects in Fig. 3b and 3c (when compared to Fig. 3a) where we similarly consider a situation with unconstrained growth (d = 0). In our model we find that growth constraints ($d>0$) advances the optimal switch time (Fig. 3d). This is in line with our simulations of the Macevicz and Oster model where the constrained model (Fig. S1d) has an earlier switch than the unconstrained model (Fig. S1a). Increasing degree of growth constraints similarly advances the optimal switch point in models of annual plants (Weis et al 2014) analogous to the Macevicz and Oster model.

In the scenario of progressive provisioning with parameters representing the *B. terrestris* life history as the starting point (Fig. 4), the optimal switch occurs later when worker mortality increase (Fig. 5a) and when worker productivity decreases (Fig. 5b). These

responses are opposite to those predicted by Macevicz and Oster (1976) for unconstrained growth and shown in Fig. S1b,c. This difference occurs because we assume that *B. terrestris* is subject to growth constraints ($d > 0$), a factor that can modulate in which direction the optimal switch time responds to variation in model parameters. For example, using a model for annual plants analogous to the Macevicz and Oster model with logistic growth (eq. S3), Lindh et al (2015) showed that decreasing productivity can delay the optimal switch to reproductive growth if the degree of growth constraint (corresponding to b_1) is sufficiently strong, similar to here (Fig. 5a). Increasing the worker mortality can delay the optimal switch point under stronger growth constraints in the Macevicz and Oster model (eq. S3) as well (as in Fig. 5b). We note, lastly, that the effects of less constrained growth (Fig. 5c) and longer season (Fig. 5d) on optimal switch times are in line with the results of the unconstrained model (Fig. S1d above and eq. 6 in Macevicz and Oster 1976, respectively) whereas body size of sexuals (with no effect on optimal switch time in Fig. 5e) is not considered in the Macevicz and Oster framework and hence not comparable.

These comparisons show that optimal switch points in our model, as a specific component of an optimal egg-laying schedule, respond to parameter variation as expected from analyses of the Macevicz and Oster (1976) model and closely related dynamic energy allocation models.

Reference

Weis, AE, Wadgymar SM, Sekor M, Franks SJ (2014) The shape of selection: using alternative fitness functions to test predictions for selection on flowering time. Evol Ecol 28: 885-904.

Appendix S3: Matlab code.

```
%NUMERICAL IMPLEMENTATION OF THE OPTIMIZATION METHODS
%FOR THE MANUSCRIPT
%"How competition between overlapping generations can influence
%optimal egg-laying strategies in annual social insects %
%By Jacob Johansson, Andres Arce, & Richard J. Gill
%
%Intended for publication in Oecologia
%
%
%CODE BY JACOB JOHANSSON 2022
%
%
%
%This file produces all data for figures 2,3,4 and 5
%the output is plotted here by the end of the chosen simulation
%(the figures in the manuscript itself were put together
%in a different order than here since they use different types of simulations)


%KEY TO VARIABLE NAMES
%(SYMBOL) DEFINITION IN PAPER = VARIABLE NAME IN THIS CODE


%  (t)      time (in weeks) = t
%  (T)      season length (weeks of egg-laying) = T_season_length
%  (r_q)    work contributed by queen = r_r_queen_productivity
%  (r_w)    work contributed per worker = r_worker_productivity
%  (s_a)    survival of adults = surv_a;
%  (d)      degree of growth constraints = d_constraints
%  (t_s)    switch point = switch_time
%  (e_a,t)  relative efficiency of a worker = e_effectivity_per_worker_v (vector)
%  (E_t)    total amount of work afforded by colony = E_total_work_afforded
%  (R_t)    colony energy income per week = R_colony_energy_income_t

%  (w_max_weight_workers)  maximum (optimal) weight of worker =
w_max_weight_workers__weight_sexuals
%  (v_max)  maximal (optimal)weight of sexual = v_max_weight_sexuals
%  (w_min_weight_workers)  minimal weight of worker to be functional =
w_min_weight_workers
%  (v_min)  minimal weight of sexual to be functional = v_min

%  (w_1)    weight/cost of 1st stage worker larvae= w_1_cost
%  (v_1)    weight/cost of 1st stage sexual larvae= v_1_cost
%  (g_max)  maximum energy demand of 2nd stage worker larvae = g_max
%  (h_max)  maximum energy demand of 2nd stage sexual larvae = h_max

%  (c_t)    egg_laying rate         = c_EGGNUM_v (vector)
%  (n_a,t)  number of worker adults = nv (vector)
%  (w_a,t)  weight of worker adults = wv (vector)
%  (m_a,t)  number of sexual adults = nsv (vector)
%  (v_a,t)  weight of sexual adults = wsv (vector)


%NOTES ABOUT THE NUMERICAL IMPLEMENTATION
%
%In relation to the model definition the following steps of the numerical
%implementation are worth noting:
%
%
%A. REPRESENTATION OF EGG-LAYING RATES
```

```
%
%
%For numerical reasons during the optimization process, egg-laying rates are
%in some cases expressed as "relative egg-laying effort", rather than "number of
eggs".
% The relative egg-laying effort is expressed by a vector "U"
%i.e. the fraction of available energy used for egg-laying per week.
%taking values between 0-1.
%
%In some analyses it is however necessary to  calculate egg-laying
%rates as "number of eggs per week", as in the model definition. These
%rates are denoted using the variable "c_EGG_NUM_v"
%so the implementation switches between these two
%ways of representing egg-laying rate when appropriate.
%
%
%CONSTRAINED OPTIMIZATION

%During the search for an optimal egg-laying schedule,the optimizer function
(fminsearch)
%updates the candidate egg-laying schedule iteratively after having evaluated its
fitness.
%It may then occur that the candidate egg-laying schedule takes on values
%that are biologically unrealistic, such as negative values or laying of more eggs
%than there are resource for. This is here addressed in two ways:
%
%
%1)The relative egg-laying effort is expressed by a parameter U
%ensures
%
%2) Penalty-functions add a fitness cost to egg-laying schedules
%that are not within biologically realistic bounds, for example
%due to rounding errors or approximations.
%

%max/min-functions (Heavyside-functions) can cause numerical problems, since
%the optimizer function (fminsearch) works best with smooth objective functions
%Therefore max-functions in the model definitions have here
%been replaced by soft min/max function where the sharpness is controlled by a
parameter K
%the soft min/max functions are denoted softJmax() and softJmin()
%respectively
%
%Parameter values of g_max = 0 and w1 = 1 can cause numerical problems, and have
%been implemented as g_max = 0.000001 and w1 =0.999999.
%
%
%
%

function main_simulator
clear all
close all

%choose which figure to show by setting the simulation_type

%Simulation_type 1
%simulation with predetermined egg laying scheduel,
%example with constant egg-laying rate with switch point at t=6
%(Figure 2AB in the manuscript)
```

```
%Simulation_type 2
%simulation of an optimal egg-laying schedule
%with fitness consequences of small deviations from the
%example with the optimal egg-laying schedule for B. terrestris settings
%(Figure 4AB in the manuscript)




%Simulation_type 3
%simulation to predict optimal egg-laying schedule
%(Figure 3ABCDE and Figures 5ABCDE in the manuscript)
%(to switch between reproducing Figure 3ABCDE or Figure 5ABCDE comment and
%uncomment the input data for the respective Figures as indicated below
%[rows 200-225])



%choice of simulation_type
simulation_type=3;


switch simulation_type


    case 1
        %SIMULATION CONTROL FOR THIS CASE
        no_reps=2; %number of times parameter settings are varied (and number of
figure columns)
        no_types_pvar=1; %number of parameters that are varied (around default
parameter values)
        egg_schedule_type=1; %U parametrized by 1-2 control parameters
        sub_model_levels=1; %no alternative submodels
        egg_measure_relative=0; %means that we measure eggs in relative egg effort 0-1

        %PARAMETERS FOR THIS CASE
        surv_var=         [1,1];
        r_worker_productivity_var= 0.5*[1,1];
        d_constraints_var=         [0,0];
        T_season_length_var=       [10,10];
        v_max_weight_sexuals_var=  [1,1];
        w_1_cost_var=       [0.3 0.9999];
        g_max_var=         [0.7 0.0001];
        switch_var =      [6, 6];
        c_EGG_NUM_v=12*[1 1 1 1 1 1 1 1 1 1 0 0 0];

    case 2
        %SIMULATION CONTROL FOR THIS CASE
        no_reps=1; %number of times parameter settings are varied (and number of
figure columns)
        no_types_pvar=1; %number of parameters that are varied (around default
parameter values)
        egg_schedule_type=0; %U free to evolve
        sub_model_levels=1; %no alternative submodels
        egg_measure_relative=1; %means that we measure eggs in relative egg effort 0-1


        %PARAMETERS FOR THIS CASE
        surv_var=         0.8;
        r_worker_productivity_var= 1.75;
        d_constraints_var=         0.01;
        T_season_length_var=         10;
        v_max_weight_sexuals_var=  2;
```

```
        w_1_cost_var=       0.3;
        g_max_var=         0.7;






    case 3
        %SIMULATION CONTROL FOR THIS CASE
        %no_reps is set further down
        no_types_pvar=1;
        egg_schedule_type=0; %U free to evolve
        sub_model_levels=1; %no alternative submodels
        egg_measure_relative=1; %means that we measure eggs in relative egg effort 0-1


        %PARAMETERS FOR THIS CASE

        %%PLEASE COMMENT AND UNCOMMENT BELOW TO SWITCH BETWEEN
        %%REPRODUCING FIGURE 3 AND FIGURE 5


        %DATA FOR FIGURE 3
        no_reps=5;

        surv_var=                       [1 1      0.8  1    0.8];
        r_worker_productivity_var=  0.5*[1 0.8    1    1    1.75/0.5];
        d_constraints_var=              [0 0      0    0.005   0.01];
        T_season_length_var=            [1 1      1    1      1]*10;
        v_max_weight_sexuals_var=       [1 1      1    1      2];
        w_1_cost_var=       0.9999*[1 1 1 1 1];
        g_max_var=          0.001*[ 1 1 1 1 1];

        %DATA FOR FIGURE 5


% %         no_reps=10;
% %
% %
% %         surv_var=         0.8 * [0.3 1/0.8 1 1 1 1 1 1 1 1];
% %         r_worker_productivity_var= 1.75 * [1 1 0.3 1.5 1 1 1 1 1 1];
% %         d_constraints_var=         0.01 * [1 1 1 1 2 0.5 1 1 1 1];
% %         T_season_length_var=         [10 10 10 10 10 10 6 14 10 10];
% %         v_max_weight_sexuals_var=  [2 2 2 2 2 2 2 2 4 1 ];
% %         w_1_cost_var=       0.3* [1 1 1 1 1 1 1 1 1 1];
% %         g_max_var=         0.7*[1 1 1 1 1 1 1 1 1 1];
% %



end


%BIOLOGICAL PARAMETERS USED FOR ALL SIMULATIONS
extraparams.r_queen_productivity=7;
extraparams.w_max_weight_workers=1;
extraparams.w_min_weight_workers=0.4;

%NOTE: Sexual body size growth parameters are set further down as follows:
%vmin = v_max_weight_sexuals*w_min_weight_workers;
%v_1_cost = v_max_weight_sexuals*w_1_cost;
%h_max = v_max_weight_sexuals*g_max;
```

```
%SIMULATION CONTROL PARAMETERS
%%%extraparams.no_rep_ix=1;
extraparams.plotting_col=1;
%%%extraparams.plotting_on=0;
%%%extraparams.verbose=0;
extraparams.fixed_egg=1;
extraparams.energy_loss_time=0;
extraparams.egg_schedule_type=egg_schedule_type;
extraparams.egg_measure_relative=egg_measure_relative;


%TREATMENT OF SIMULATION TYPE 1 (NO OPTIMIZATION)
%for simulation type 1 we are not optimizing, only running the demographic
%model for fixed constant egg-laying
if simulation_type==1
    for jpara = 1:no_reps

        %setting of parameter values for each colony simulation
        extraparams.surv_a=surv_var(jpara);
        extraparams.r_worker_productivity=r_worker_productivity_var(jpara);
        extraparams.d_constraints=d_constraints_var(jpara);
        extraparams.T_season_length=T_season_length_var(jpara);
        extraparams.v_max_weight_sexuals=v_max_weight_sexuals_var(jpara);
        extraparams.w_1_cost=w_1_cost_var(jpara);
        extraparams.g_max=g_max_var(jpara);
        extraparams.switch_time=switch_var(jpara);

        %parameters controlling body size growth of sexuals scale
        %with queen_weight
        extraparams.vmin =
extraparams.v_max_weight_sexuals*extraparams.w_min_weight_workers;
        extraparams.v_1_cost = extraparams.v_max_weight_sexuals*extraparams.w_1_cost;
        extraparams.h_max = extraparams.v_max_weight_sexuals*extraparams.g_max;

        extraparams.K=100;

        extraparams.submodeltype=6;
        extraparams.fixed_egg=1;

[fitness_out(jpara),outv(1,1,jpara).out,pv(1,1,jpara).p]=fitnessfun(c_EGG_NUM_v,extrap
arams);
    end
    plot_demography_sim_verbose_embedded(outv,pv)

    fitness_out
    disp('plot done, end')
    pause


end

%TREATMENT OF SIMULATION TYPE 2 AND 8 (OPTIMIZATION)

%reference_T_season_length = reference value for optimization of switch times,
%and used to adjust the vector for switch times to be probed if season
%lengths changes
%needs to be an even number
extraparams.reference_T_season_length=10;
```

```
%LOOP 1 runs over the number of parameter settings (each resulting in one optimal egg-
laying schedule)


%LOOP 2 runs over different types of constrained models
%CONTROL OF LOOP 2
numcontrolparamsv=[1,2,3,1,2,1];
fixeggv=[1,1,1,0,0,1];


%CONTROL OF LOOP 3
%LOOP 3 runs over the switch times to be tested for optimality
%Each iteration starts with uniform egg-laying strategy as initial state
switch_vec=[3 4 5 6 7 8]; %candidate switch times to be tested for optimality
s_levels=1:6; %indexes of candidate switch times

%CONTROL OF LOOP 4
%LOOP 4 runs over increasingly sharp soft max/min function
%The first iteration starts with uniform egg-laying strategy as initial state
%Later iterations starts with the optimal strategy from the previous
%iteration, so as to successively refine the solution.
Kv=5*[20 20 20 80 20 20 20 320 640 1280 2000];
K_levels=1:3;


%LOOP 1 over the number of parameters to be varied
for ipara=1:no_types_pvar
    %LOOP 1 over the number of parameter settings (each resulting in one optimal egg-
laying schedule)
    for jpara=1:no_reps %resolution (variation in each parameter)

        %ipara
        disp(['Optimization under parameter set',num2str(jpara)])

        %setting of parameter values for each optimization
        extraparams.surv_a=surv_var(jpara);
        extraparams.r_worker_productivity=r_worker_productivity_var(jpara);
        extraparams.d_constraints=d_constraints_var(jpara);
        extraparams.T_season_length=T_season_length_var(jpara);
        extraparams.v_max_weight_sexuals=v_max_weight_sexuals_var(jpara);
        extraparams.w_1_cost=w_1_cost_var(jpara);
        extraparams.g_max=g_max_var(jpara);

        %parameters controlling body size growth of sexuals scale
        %with queen_weight
        extraparams.vmin =
extraparams.v_max_weight_sexuals*extraparams.w_min_weight_workers;
        extraparams.v_1_cost = extraparams.v_max_weight_sexuals*extraparams.w_1_cost;
        extraparams.h_max = extraparams.v_max_weight_sexuals*extraparams.g_max;



        %LOOP 2 over different constrained egg-laying models
        for sub_model_type=sub_model_levels

            %starting conditions for egg_laying vectors (to be optimized)
            %egg-laying schedule freely evolving
            Ustart00=extraparams.w_1_cost*ones(1,(extraparams.T_season_length));
            %egg-laying schedule constrained by alternative model
            ControlParamStart00=ones(1,numcontrolparamsv(sub_model_type));

            extraparams.submodeltype=sub_model_type;
            extraparams.fixed_egg=fixeggv(sub_model_type);
```

```
            %LOOP 3 runs over the switch times to be tested for optimality
            for ss=s_levels
                %adjust the vector for switch times to be probed if season
                %lengths changes
                switch_range_adjust=(extraparams.T_season_length-
extraparams.reference_T_season_length)/2; %adjustment
                extraparams.switch_time=switch_vec(ss)+ switch_range_adjust;%

                %intiate egg_laying vectors
                Ustart=Ustart00;
                ControlParamStart=ControlParamStart00;


                %LOOP 4 runs over increasing sharp soft max/min function
                for kkk=K_levels
                    extraparams.K=Kv(kkk);

                    %egg-laying strategy parametrized by 1-2 control parameters
                    if egg_schedule_type==0
                        extraparams.K=Kv(kkk);
                        fitfun=@(U) fitnessfun(U,extraparams)

                        [predicted_strategy,fval,exitflag,output]
=fminsearch(fitfun,Ustart);
                        Ustart=predicted_strategy;


                        %egg-laying strategy parametrized by 1-2 control parameters
                    elseif egg_schedule_type==1
                        fitfun=@(ccpp) fitnessfun(ccpp,extraparams)
                        [predicted_strategy,fval,exitflag,output]
=fminsearch(fitfun,ControlParamStart);
                        CP=predicted_strategy;
                    end



                end %END LOOP 4 over K

                fitness_switch(ss)=-fval;
                switch_time_realised(ss)=extraparams.switch_time;
                CPstart_vec(ss,1:length(predicted_strategy))=predicted_strategy;

            end %END LOOP 3 over switch times



            %find the optimal switch time
            [val,optimal_switch_time_index]=max(fitness_switch);

optimal_switch_time(sub_model_type)=switch_vec(optimal_switch_time_index)+switch_range
_adjust;

fitness_treatment(sub_model_type)=fitness_switch(optimal_switch_time_index);

            %retrieve the corresponding strategy and store it

predicted_strategy_opt_switch=CPstart_vec(optimal_switch_time_index,1:length(predicted
_strategy));

predicted_strategy_v(sub_model_type,1:length(predicted_strategy))=predicted_strategy_o
pt_switch;
```

```
            extraparams.switch_time=optimal_switch_time(sub_model_type);

            %run the corresponding strategy

[fitness_tr(sub_model_type),outv(sub_model_type).out,pv(sub_model_type).p]=fitnessfun(
predicted_strategy_opt_switch,extraparams)

            %store its dynamics (stored in outv) and along with all
            %parameters (stored in pv)
            fitness_tr(sub_model_type)=-fitness_tr(sub_model_type); %make fitness
positive (since fminsearch finds the minimum)
            fitness_tot(sub_model_type,ipara,jpara)=fitness_tr(sub_model_type);
            outv_tot(sub_model_type,ipara,jpara).out=outv(sub_model_type).out;
            pv_tot(sub_model_type,ipara,jpara).p=pv(sub_model_type).p;


            %keep track of fitness of all probed switch times for later
            %plotting
            switch_monitor(sub_model_type,ipara,jpara).fs=fitness_switch;
            switch_monitor(sub_model_type,ipara,jpara).str=switch_time_realised;

        end %END LOOP 2 over submodel types

    end %END LOOP 1 over jpara, parameter settings

end  %END LOOP 0 over ipara, parameter types



%handling of data for plotting
switch simulation_type




    case 1

        plot_demography_sim_verbose_embedded(outv_tot,pv_tot)

    case 2

        plot_demography_sim_verbose_embedded(outv_tot,pv_tot)

        %PREPARE FOR FITNESS SENSITIVITY CHECK
        %retrive the optimal strategy
        opt_strat_c_EGG_NUM_v=outv_tot.out.c_EGG_NUM_laid
        %retrieve the associated resource supply function
        opt_strat_res_supply=outv_tot.out.res_supply

        %run the fitness sensitivity function (to produce Fig 3C)

fitness_sensitivity_fun(opt_strat_res_supply,opt_strat_c_EGG_NUM_v,extraparams);

        %RUN TWO EXEMPLARY SIMULATIONS WITH REDUCED EGG-LAYING (to produce Fig 3DE)
        extraparams.egg_measure_relative=0; %means we will use the fixed eggs here
(otherwise all egg-laying rates may change!)
        extraparams.fixed_egg=1;
        extraparams.egg_schedule_type=1;
        extraparams.submodeltype=6;

        %reduce by 50% at two time points
        strategy_perturbed_early=opt_strat_c_EGG_NUM_v;
        strategy_perturbed_early(1)=strategy_perturbed_early(1)/2;
```

```
        strategy_perturbed_late=opt_strat_c_EGG_NUM_v;
        strategy_perturbed_late(9)=strategy_perturbed_late(9)/2;

        %simulate and plot
        fitnessfun(opt_strat_c_EGG_NUM_v,extraparams);

[fitn,example_outv(1).out,example_pv(1).p]=fitnessfun(strategy_perturbed_early,extrapa
rams);

[fitn,example_outv(2).out,example_pv(2).p]=fitnessfun(strategy_perturbed_late,extrapar
ams);

        for iii=1:2
            res_supply=example_outv(iii).out.res_supply;
            res_demand=example_outv(iii).out.res_demand;
            time_v=linspace(1,example_pv(iii).p.T_end_SIM,example_pv(iii).p.T_end_SIM)

            figure(33)
            subplot(1,2,iii)
            hold on
            plot(time_v,res_supply,'m-','linewidth',2)
            plot(time_v,res_demand,'k:','linewidth',2)

            %set(gca,'ylim',[0,45])
            box off
            %ylabel('supply & demand');
            %xlabel('time (weeks)')
        end

    case 3

        plot_demography_sim_verbose_embedded(outv_tot,pv_tot)


end



disp('done ****')



function fitness_sensitivity_fun(res_supply,egg_laying_reference,extraparams)

%fitness landscape
figure(40)

%for a fixed egg schedule measured in egg laying rate, not effort.
extraparams.egg_measure_relative=0; %means we will use the fixed egg laying rates here
(otherwise all egg-laying rates may change!)
extraparams.fixed_egg=1; %dito
extraparams.egg_schedule_type=1; %
extraparams.submodeltype=6;
extraparams.K=40;
extraparams.plotting_on=0;
Ucandstartv=linspace(0,1,50);
c_EGG_NUM_reference=[egg_laying_reference];

for j=1:10
    disp(['Fitness sensitivity for egg laid in week: ',num2str(j)])

    %for each week egg-laying rates are varied
    %between 0 and maximum possible number of eggs laid
```

```
    %the maximum is given by:
    c_EGG_NUM_max=res_supply(j)/extraparams.w_1_cost;


    for i=1:50

        c_EGG_NUM_candstart=Ucandstartv(i)*c_EGG_NUM_max;
        extraparams.UFIXBEFORE=c_EGG_NUM_reference(1:(j-1));
        extraparams.UFIXAFTER=c_EGG_NUM_reference((j+1):11);

c_EGG_NUM0v_cand=[extraparams.UFIXBEFORE,c_EGG_NUM_candstart,extraparams.UFIXAFTER];


        [fitn,outv(i).out,pv(i).p]=fitnessfun(c_EGG_NUM0v_cand,extraparams);
        fitness_perturbed_v(i)=-fitn;
        number_eggs_v(i)=outv(i).out.c_EGG_NUM_laid(j);
    end


    [fitn,outvfix.out,pvfix.p]=fitnessfun(c_EGG_NUM_reference,extraparams);
    fitness_reference=-fitn;
    number_eggs_reference=outvfix.out.c_EGG_NUM_laid(j);


    hold on
    subplot(2,5,j)
    plot(number_eggs_v,fitness_perturbed_v,'b','linewidth',1)
    hold on
    plot(number_eggs_reference,fitness_reference,'r*')
    box off
%      %set x-axis to have the same scale of 20 points
%      if (number_eggs_reference-10)<0
%          xmin=0;
%          xmax=20;
%
%      else
%          xmin=number_eggs_reference-10;
%          xmax=number_eggs_reference+10;
%      end
    xmin=0;
    xmax=50;
    set(gca,'xlim',[xmin,xmax])


    set(gca,'ylim',[max(fitness_perturbed_v)-80 max(fitness_perturbed_v)+20])

    if j==1 | j==6
        ylabel('fitness');

    else
        set(gca,'yticklabels',[])
    end
    title(['t = ',num2str(j)])
end


%function that returns the fitness of a specific egg-laying schedule
function [fitness,out,p]=fitnessfun(candidate_strategy,extraparams)


%unpacking extraparams and put them in the p. struct
p.switch_time=extraparams.switch_time;
%p.verbose=extraparams.verbose;
```

```
p.fixed_egg=extraparams.fixed_egg;
p.submodeltype=extraparams.submodeltype;
p.egg_measure_relative=extraparams.egg_measure_relative;

if extraparams.egg_schedule_type==0; %freely evolving U
    Ucand=candidate_strategy(1:end);
elseif extraparams.egg_schedule_type==1 %alternative strategies
    CP=candidate_strategy;
    p.CP=CP;


    %construct the egg-laying rates
    EggcandZeros=zeros(1,(extraparams.T_season_length));
    switch extraparams.submodeltype
        case 1 %constant egg-laying rate
            Eggcand = CP(1)*1+EggcandZeros;
        case 2
            Eggcand=EggcandZeros;
            Eggcand(1: extraparams.switch_time-1) = CP(1);
            Eggcand(extraparams.switch_time:end) = CP(2);
        case 3
            Eggcand=EggcandZeros;
            Eggcand(1) = 1+CP(1);
            Eggcand(3) = CP(2);
            Eggcand(5:end) = CP(3);
        case 4
            Eggcand=NaN(size(EggcandZeros));

        case 5
            Eggcand=NaN(size(EggcandZeros));
        case 6 %used for a given test strategy
            Eggcand=candidate_strategy;


    end
    Eggcand=[Eggcand,0,0,0];

end


%unpacking extraparameters for plotting and control
p.K=extraparams.K;
p.T_end_SIM=extraparams.T_season_length+3; %colony cycle length, time in days between
colony start and switch point

%default values of parameters p. (but which may change in different settings)
p.surv_a=extraparams.surv_a; %adult survival
p.surv_p=1; %pupal survival

p.d_constraints=extraparams.d_constraints; %competitive strength
p.v_max_weight_sexuals=extraparams.v_max_weight_sexuals;
p.w_min_weight_workers=extraparams.w_min_weight_workers; %minimal worker size to be
useful
p.w_1_cost=extraparams.w_1_cost; % extraparams.w_1_cost; % energetic cost of an egg

p.w_max_weight_workers=extraparams.w_max_weight_workers;
p.g_max=extraparams.g_max;
p.vmin=extraparams.vmin ;
p.v_1_cost=extraparams.v_1_cost;
p.h_max=extraparams.h_max;

%pupation and eclosion ages
p.pup_age=2;
```

```
p.ecl_age=3;





p.r_queen_productivity=extraparams.r_queen_productivity;
p.r_worker_productivity=extraparams.r_worker_productivity;

%energy loss time is by default 0 but is used in some analysis
p.energy_loss_time=extraparams.energy_loss_time;

p.initial_number_workers=0;
p.initial_pupae=0;
p.initial_weight=0;



if extraparams.egg_schedule_type==0; %freely evolving U

    %penalties to ensure the egg-laying rates and switch time are within
    %bounds (e.g. remain positive) specified by the model

    error_switch_below_1=min(p.switch_time,1)-1;
    error_above_season_length=max(p.switch_time,extraparams.T_season_length)-
extraparams.T_season_length;
    total_error_switch=abs(error_switch_below_1)+abs(error_above_season_length);

    error_below_zero=min(zeros(size(Ucand)),Ucand);
    error_above_one=max(ones(size(Ucand)),Ucand)-1;
    %error_egg_cost_late=sum(abs(Ucand((p.T_end_SIM-1):p.T_end_SIM+1)));
    total_error_U=sum(abs(error_below_zero)+abs(error_above_one));
%+error_egg_cost_late;

    penalty=total_error_U*10e6+total_error_switch*100;

    %restirct Ucand to between 0 and 1.
    if any(Ucand>1)
        Ucand(Ucand>1)=1;
    end

    if any(Ucand<0)
        Ucand(Ucand<0)=0;
    end

    p.U0v=[Ucand,0,0,0];
    p.Egg0v=p.U0v;

elseif extraparams.egg_schedule_type==1; %alt strategy
    %*********
    if p.fixed_egg==1 %
        error_switch_below_1=min(p.switch_time,1)-1;
        error_above_season_length=max(p.switch_time,extraparams.T_season_length)-
extraparams.T_season_length;

        total_error_switch=abs(error_switch_below_1)+abs(error_above_season_length);

        error_below_zero=min(zeros(size(Eggcand)),Eggcand);
        error_above_one=max(ones(size(Eggcand)),Eggcand)-1;
        %   error_egg_cost_late=sum(abs(Eggcand((p.T_end_SIM-1):p.T_end_SIM+1)));
        total_error_U=sum(abs(error_below_zero));%+error_egg_cost_late;

        penalty=total_error_U*10e6+total_error_switch*100;
```

```
    else
        penalty=0;
        error_below_zero=min(zeros(size(p.CP)),p.CP);
        total_error=sum(abs(error_below_zero));%+error_egg_cost_late;
        penalty=total_error*10e6;
    end

    if any(Eggcand<0)
        Eggcand(Eggcand<0)=0;
    end

    p.Egg0v=[Eggcand,0,0,0];
    Egg0fix=p.Egg0v;

end

out=simulate_demography_master(p);
fitness_out=out.colony_fitness;

%adding penalties to fitness function (- since fminsearch looks for a minimum)
fitness=-1*(fitness_out-penalty);


%function simulating the colony demographic model
function out=simulate_demography_master(p)


%WORKER MATRICES
n_time=p.T_end_SIM;
n_all_ages=n_time; %could potentially be longer than n_time if you seeded the
population with an old cohort


%pre-allocation of state variable vectors
%worker numbers
nv=zeros(n_all_ages,1); %numbers per age class for current time step
nv_old=zeros(n_all_ages,1); %numbers per age class previous time step
nv_tot=zeros(n_all_ages,n_all_ages); %numbers per age class all time steps

%worker weights
wv=zeros(n_all_ages,1); %weights per age class for current time step
wv_old=zeros(n_all_ages,1); %weights per age class previous time step
wv_tot=zeros(n_all_ages,n_all_ages); %weights per age class all time steps

%sexual numbers (same structure as nv)
nsv=zeros(n_all_ages,1);
nsv_old=zeros(n_all_ages,1);
nsv_tot=zeros(n_all_ages,n_all_ages);

%sexual weights (same structure as wv)
wsv=zeros(n_all_ages,1);
wsv_old=zeros(n_all_ages,1);
wsv_tot=zeros(n_all_ages,n_all_ages);


%pre-allocation of plotting vectors
n_adults_tot=zeros(1,n_time); %vectors for monitoring total numbers
c_tot=zeros(1,n_time);
n_larvae_tot=zeros(1,n_time);

n_s_adults_tot=zeros(1,n_time); %vectors for monitoring total numbers
n_s_pupae_tot=zeros(1,n_time);
n_s_larvae_tot=zeros(1,n_time);
```

```
biomass_adults_tot=zeros(1,n_time); %vectors for monitoring total biomass
biomass_pupae_tot=zeros(1,n_time);
biomass_larvae_tot=zeros(1,n_time);

biomass_s_adults_tot=zeros(1,n_time); %vectors for monitoring total biomass
biomass_s_pupae_tot=zeros(1,n_time);
biomass_s_larvae_tot=zeros(1,n_time);

mean_adult_size_at_eclosion=zeros(1,n_time); %vectors for monitoring body sizes
mean_s_adult_size_at_eclosion=zeros(1,n_time);

res_supply=zeros(1,n_time);
res_demand=zeros(1,n_time);
c_EGG_NUM_laid=zeros(1,n_time);
larval_growth_v=zeros(1,n_time);



%%%%%%%%%%%%%%%%%%%%


time_v=linspace(1,p.T_end_SIM,n_all_ages);


nv(p.ecl_age)=p.initial_number_workers;
wv(p.ecl_age)=p.initial_weight;



%LOOP over weeks in the colony cycle
for t=1:n_time




    %%%%DYNAMICS
    %CALCULATE THE COLONY DEMAND

    num_larvae=sum(nv(1)+nsv(1)); %number of growing worker larvae (i.e. the eggs of
last week)


    %CALCULATE THE COLONY INCOME


    %equation 3
    e_effectivity_per_worker_v=softJmax(0,wv(p.ecl_age:n_all_ages)-
p.w_min_weight_workers,p.K)/(p.w_max_weight_workers-p.w_min_weight_workers);

    %second term in equation 4
    E_effectivity_of_all_workers=
e_effectivity_per_worker_v'*nv(p.ecl_age:n_all_ages);

    %equation 4
    E_total_work_afforded = p.r_queen_productivity +
p.r_worker_productivity*E_effectivity_of_all_workers;

    %equation 5

R_colony_energy_income_t=E_total_work_afforded/(1+p.d_constraints*E_total_work_afforde
d);
```

```
    %energy loss used in some analyses, but not as default
    if t==p.energy_loss_time
        R_colony_energy_income_t=R_colony_energy_income_t-1;
    end

    %GROWTH RATES OF ALL LARVAE AS WELL AS THE INITIAL INVESTEMENT IN
    %EGGS (GROWTH DAY 1)

    %colony maximum demand (assumed to be proportional to #larvae + eggs which also
need an initial size)

    if t<p.switch_time %before the switch we have worker eggs, at switch_time and
after we have sexual eggs
        w_1_cost_wq=p.w_1_cost;
    else
        w_1_cost_wq=p.v_max_weight_sexuals*p.w_1_cost;
    end

    if t<(p.switch_time+1) %the sexual 2nd stage larvae appear at switch_time+1
        p.max_larval_demand=p.g_max;
    else
        p.max_larval_demand=p.h_max;
    end

    max_demand_per_day=p.max_larval_demand*num_larvae;



    if p.egg_measure_relative

        egg_effort = p.Egg0v(t);
        eggs_realised_per_day=egg_effort*R_colony_energy_income_t/w_1_cost_wq;

    else


        if p.fixed_egg==1
            number_eggs = p.Egg0v(t);


        elseif p.submodeltype==4
            if t<(p.T_end_SIM-2)
                number_eggs=p.CP(1)*R_colony_energy_income_t;
            else
                number_eggs=0;
            end
        elseif p.submodeltype==5
            if t<(p.T_end_SIM-2)
                number_eggs=p.CP(1)+p.CP(2)*(sum(nv(2)+nsv(2))); %so those that will
be pupae next week
            else
                number_eggs=0;
            end
        end

        %check if there are sufficient supply to
        %to lay the intended amount of eggs
        %if not adjust number of eggs to fit supply

        if R_colony_energy_income_t/p.w_1_cost>number_eggs;
            eggs_realised_per_day=number_eggs;
```

```
        egg_effort=eggs_realised_per_day*w_1_cost_wq/R_colony_energy_income_t;
    else
        eggs_realised_per_day=R_colony_energy_income_t/w_1_cost_wq;
        egg_effort=1;
    end

end

%calculate how much supply is left for larval growth
%after eggs have been supplied
supply_for_larvae=R_colony_energy_income_t-eggs_realised_per_day*w_1_cost_wq;


%error check: are number of larvae negative?
if num_larvae<=0
    larval_growth=0;
    supply_for_larvae=0;
    if num_larvae<-0.01,
        num_larvae
        eggs_realised_per_day
        egg_effort
        R_colony_energy_income_t
        p
        warning('num_larvae negative'),
        pause
    end
else
    %calculate the per larvae body size growth
    larval_growth=softJmin(p.max_larval_demand,supply_for_larvae/num_larvae,p.K);

end



%AGING AND GROWTH (=moving forward one time step)

%current generation becomes the previous generation
nv_old=nv;
wv_old=wv;
nsv_old=nsv;
wsv_old=wsv;

%aging and growth of larvae
nv(2)=nv_old(1);
wv(2)=wv_old(1)+larval_growth;
nsv(2)=nsv_old(1);
wsv(2)=wsv_old(1)+larval_growth;

%aging for adults and pupae
nv(3:n_all_ages)=nv_old(2:(n_all_ages-1));
wv(3:n_all_ages)=wv_old(2:(n_all_ages-1));
nsv(3:n_all_ages)=nsv_old(2:(n_all_ages-1));
wsv(3:n_all_ages)=wsv_old(2:(n_all_ages-1));

%survival pupae  %observe: no transport here
nv(3)=(1-(1-p.surv_p))*nv(3);
nsv(3)=(1-(1-p.surv_p))*nsv(3);

%survival adults  %observe: no transport here
nv(4:n_all_ages)=(1-(1-p.surv_a))*nv(4:n_all_ages);
nsv(4:n_all_ages)=nsv(4:n_all_ages); %no mortality here
```

```
    %births (note these first-entry elements needs to be added last,
    %because otherwise they are transported to next age-class
    %as the larvae grows a few code lines above)


    if t<p.switch_time
        nv(1)=eggs_realised_per_day;
        wv(1)=w_1_cost_wq;
        nsv(1)=0;
        wsv(1)=0;
    else
        nv(1)=0;
        wv(1)=0;
        nsv(1)=eggs_realised_per_day;
        wsv(1)=w_1_cost_wq;
    end





    %%%%END DYNAMICS



    %%%%PLOTTING & SAVING

    %saving output for plotting and analyses

    nv_tot(1:n_all_ages,t)=nv;
    wv_tot(1:n_all_ages,t)=wv;
    nsv_tot(1:n_all_ages,t)=nsv;
    wsv_tot(1:n_all_ages,t)=wsv;


    nv_adults = nv(p.ecl_age:n_all_ages);
    nv_pupae  = nv(p.pup_age:(p.ecl_age-1));
    nv_larvae = nv(1:(p.pup_age-1));

    nv_s_adults = nsv(p.ecl_age:n_all_ages);
    nv_s_pupae  = nsv(p.pup_age:(p.ecl_age-1));
    nv_s_larvae = nsv(1:(p.pup_age-1));

    wv_adults = wv(p.ecl_age:n_all_ages);
    wv_pupae  = wv(p.pup_age:(p.ecl_age-1));
    wv_larvae = wv(1:(p.pup_age-1));

    wv_s_adults = wsv(p.ecl_age:n_all_ages);
    wv_s_pupae  = wsv(p.pup_age:(p.ecl_age-1));
    wv_s_larvae = wsv(1:(p.pup_age-1));

    n_adults_tot(t) = sum(nv_adults);
    n_pupae_tot(t)  = sum(nv_pupae);
    n_larvae_tot(t) = sum(nv_larvae);

    ns_adults_tot(t) = sum(nv_s_adults);
    ns_pupae_tot(t)  = sum(nv_s_pupae);
    ns_larvae_tot(t) = sum(nv_s_larvae);

    n_new_LPA(1:6,t) = [nv(1); nv(p.pup_age+1);nsv(p.ecl_age+1);nv(1);
nsv(p.pup_age+1);nsv(p.ecl_age+1)];

    biomass_tot(t) = wv' *nv;
```

```
    biomass_adults_tot(t) = wv_adults' *nv_adults;
    biomass_pupae_tot(t)  = wv_pupae'  *nv_pupae;
    biomass_larvae_tot(t) = wv_larvae' *nv_larvae;

    biomass_s_tot(t) = wsv' *nsv;
    biomass_s_adults_tot(t) = wv_s_adults' *nv_s_adults;
    biomass_s_pupae_tot(t)  = wv_s_pupae'  *nv_s_pupae;
    biomass_s_larvae_tot(t) = wv_s_larvae' *nv_s_larvae;

    %calculate fitness
    fitness_reproductives(t) = softJmax(wv_s_adults-
p.v_max_weight_sexuals*p.w_min_weight_workers,0,p.K)'
*nv_s_adults/(p.w_max_weight_workers-p.w_min_weight_workers);

    %set mean adult size to NaN if there are no individuals for plotting
    %(the body sizes are calculated regardless)

    if nv(p.ecl_age)>0.5
        mean_adult_size_at_eclosion(t)=wv(p.ecl_age);
    else
        mean_adult_size_at_eclosion(t)=NaN;
    end

    if nsv(p.ecl_age)>0.5
        mean_s_adult_size_at_eclosion(t)=wsv(p.ecl_age);
    else
        mean_s_adult_size_at_eclosion(t)=NaN;
    end

    res_supply(t)=  R_colony_energy_income_t;
    res_demand(t)= max_demand_per_day+eggs_realised_per_day*w_1_cost_wq;

    c_EGG_NUM_laid(t)=eggs_realised_per_day;
    egg_effort_v(t)=egg_effort;
    larval_growth_v(t)=larval_growth;



end %END of LOOP over weeks in the colony cycle


out.colony_fitness=fitness_reproductives(end);



%packing results and plot variables

out.n_adults_tot=n_adults_tot;
out.n_pupae_tot=n_pupae_tot;
out.n_larvae_tot=n_larvae_tot;

out.ns_adults_tot=ns_adults_tot;
out.ns_pupae_tot=ns_pupae_tot;
out.ns_larvae_tot=ns_larvae_tot;

out.n_new_LPA=n_new_LPA;

out.biomass_tot=biomass_tot;
out.biomass_adults_tot=biomass_adults_tot;
out.biomass_pupae_tot=biomass_pupae_tot;
out.biomass_larvae_tot=biomass_larvae_tot;

out.biomass_s_tot=biomass_s_tot;
```

```
out.biomass_s_adults_tot=biomass_s_adults_tot;
out.biomass_s_pupae_tot=biomass_s_pupae_tot;
out.biomass_s_larvae_tot=biomass_s_larvae_tot;

out.mean_adult_size_at_eclosion=mean_adult_size_at_eclosion;
out.mean_s_adult_size_at_eclosion=mean_s_adult_size_at_eclosion;

out.res_supply=res_supply;
out.res_demand=res_demand;
out.c_EGG_NUM_laid=c_EGG_NUM_laid;
out.larval_growth_v=larval_growth_v;
out.egg_effort_v=egg_effort_v;



%soft min/max functions to avoid numerical problems
%when optimizing body sizes
function m=softJmax(A,B,K)
m=(A.*exp(K*A)+B.*exp(K*B))./(exp(K*A)+exp(K*B));

function m=softJmin(A,B,K)
m=-(-A.*exp(-K*A)-B.*exp(-K*B))./(exp(-K*A)+exp(-K*B));

function plot_demography_sim_verbose_embedded(outv,pv)

[sm_types,leni_no_param_types,lenj_no_levels]=size(outv)
no_rep=lenj_no_levels %p.no_rep_ix; %call it no_rep for simplity, we only vary ix
here.

no_fig_rows_tot=4;

if sm_types==1
    no_fig_col_tot=leni_no_param_types*lenj_no_levels
elseif sm_types>1
    no_fig_col_tot=sm_types;
end
for smi=1:sm_types
    for ipara=1:leni_no_param_types
        for jpara=1:lenj_no_levels

            p=pv(smi,ipara,jpara).p

            %UNPACKING VARIABLES

            n_adults_tot=outv(smi,ipara,jpara).out.n_adults_tot;
            n_pupae_tot=outv(smi,ipara,jpara).out.n_pupae_tot;
            n_larvae_tot=outv(smi,ipara,jpara).out.n_larvae_tot;
            biomass_tot=outv(smi,ipara,jpara).out.biomass_tot;
            biomass_adults_tot=outv(smi,ipara,jpara).out.biomass_adults_tot;
            biomass_pupae_tot=outv(smi,ipara,jpara).out.biomass_pupae_tot;
            biomass_larvae_tot=outv(smi,ipara,jpara).out.biomass_larvae_tot;

mean_adult_size_at_eclosion=outv(smi,ipara,jpara).out.mean_adult_size_at_eclosion;

            ns_adults_tot=outv(smi,ipara,jpara).out.ns_adults_tot;
            ns_pupae_tot=outv(smi,ipara,jpara).out.ns_pupae_tot;
            ns_larvae_tot=outv(smi,ipara,jpara).out.ns_larvae_tot;
            biomass_s_tot=outv(smi,ipara,jpara).out.biomass_s_tot;
            biomass_s_adults_tot=outv(smi,ipara,jpara).out.biomass_s_adults_tot;
            biomass_s_pupae_tot=outv(smi,ipara,jpara).out.biomass_s_pupae_tot;
            biomass_s_larvae_tot=outv(smi,ipara,jpara).out.biomass_s_larvae_tot;

mean_s_adult_size_at_eclosion=outv(smi,ipara,jpara).out.mean_s_adult_size_at_eclosion;
```

```
n_new_LPA=outv(smi,ipara,jpara).out.n_new_LPA;

res_supply=outv(smi,ipara,jpara).out.res_supply;
res_demand=outv(smi,ipara,jpara).out.res_demand;
c_EGG_NUM_laid=outv(smi,ipara,jpara).out.c_EGG_NUM_laid;
larval_growth_v=outv(smi,ipara,jpara).out.larval_growth_v;

egg_effort_v=outv(smi,ipara,jpara).out.egg_effort_v;

%construct oviposition rate vectors for sexuals and workers
egg_w=zeros(size(c_EGG_NUM_laid));
egg_w(1:(p.switch_time-1))=c_EGG_NUM_laid(1:(p.switch_time-1));
egg_s=zeros(size(c_EGG_NUM_laid));
egg_s(p.switch_time:end)=c_EGG_NUM_laid(p.switch_time:end)
egg_w_effort=zeros(size(egg_effort_v));
egg_w_effort(1:(p.switch_time-1))=egg_effort_v(1:(p.switch_time-1));
egg_s_effort=zeros(size(egg_effort_v));
egg_s_effort(p.switch_time:end)=egg_effort_v(p.switch_time:end)


time_v=linspace(1,p.T_end_SIM,p.T_end_SIM)


time_v0=[0,time_v]; % for whenever eggs are involved
egg_w0=[egg_w, 0];
egg_s0=[egg_s, 0];
egg_w_effort0=[egg_w_effort,0];
egg_s_effort0=[egg_s_effort,0];
n_larvae_tot0=[0,n_larvae_tot];
n_pupae_tot0=[0,n_pupae_tot];
n_adults_tot0=[0,n_adults_tot];

ns_larvae_tot0=[0,ns_larvae_tot];
ns_pupae_tot0=[0,ns_pupae_tot];
ns_adults_tot0=[0,ns_adults_tot];


figure(20)

for i_row=1:no_fig_rows_tot

    %fig number
    if sm_types==1
        top_fig_no=(ipara-1)*lenj_no_levels+jpara
        subfigno=top_fig_no+(i_row-1)*no_fig_col_tot
    elseif sm_types>1
        top_fig_no=smi;
        subfigno=top_fig_no+(i_row-1)*no_fig_col_tot

    end
    subplot(no_fig_rows_tot,no_fig_col_tot,subfigno)


    ylabel('# eggs');
    %set(gca,'ylim',[0,40]);
    set(gca,'xlim',[-1,14]);
    BW=0.5;
    switch i_row

        case 1
            hold on
            bar(time_v0,egg_w0,'b','BarWidth',BW,'EdgeColor','None')
```

```
                        bar(time_v0,egg_s0,'r','BarWidth',BW,'EdgeColor','None')


                        set(gca,'ylim',[0,max([egg_w0,egg_s]*1.2)])

                        box off
                        ylabel('# eggs');

                    case 2

g=bar(time_v0,[n_adults_tot0;ns_adults_tot0]','grouped','BarWidth',3.5*BW)
                        g(1).FaceColor='b';
                        g(2).FaceColor='r';
                        g(1).EdgeColor='none';
                        g(2).EdgeColor='none';



                        set(gca,'ylim',[0,max([n_adults_tot0,ns_adults_tot0])*1.2])
                        box off
                        ylabel('# adults')
                    case 3
                        hold on
                        plot(time_v,res_supply,'m-','linewidth',2)
                        plot(time_v,res_demand,'k:','linewidth',2)

                        %set(gca,'ylim',[0,45])
                        box off
                        ylabel('supply & demand');
                    case 4
                        hold on
                        plot(time_v,mean_adult_size_at_eclosion,'bo','linewidth',2)
                        plot(time_v,mean_s_adult_size_at_eclosion,'ro','linewidth',2)

                        set(gca,'ylim',[0,2.2])
                        box off
                        ylabel('adults sizes')


                end


                set(gca,'xlim',[-0.5,max(time_v)+0.5])

                if top_fig_no==1
                else
                    %set(gca,'yticklabels',[])
                end

                if i_row==no_fig_rows_tot
                    %for this bottom row, show time
                    xlabel('time (weeks)');
                else
                    %set(gca,'xticklabels',[])
                end



            end

        end
    end
end
```
